# Omalizumab-Associated Post-Injection Urticaria Exacerbation and Urticaria-Related Adverse Reactions During Treatment for Chronic Urticaria: A Scoping Review

**DOI:** 10.3390/life16071124

**Published:** 2026-07-06

**Authors:** Weeratian Tawanwongsri, Pitchaya Jaruvijitrattana, Chime Eden

**Affiliations:** 1 Division of Dermatology, Department of Internal Medicine, School of Medicine, Walailak University, Nakhon Si Thammarat 80160, Thailand; 2 Center of Excellence in Data Science for Health Study, Walailak University, Nakhon Si Thammarat 80160, Thailand; 3 Division of Dermatology, Department of Internal Medicine, Chaophya Abhaibhubejhr Hospital, Prachin Buri 25000, Thailand; 4 Department of Dermatology, Jigme Dorji Wangchuck National Referral Hospital (JDWNRH), Thimphu 11001, Bhutan

**Keywords:** omalizumab, chronic spontaneous urticaria, chronic urticaria, urticaria exacerbation, post-injection reaction, adverse drug reaction, hypersensitivity, angioedema, anaphylaxis, scoping review

## Abstract

Background/Objectives: Omalizumab is an effective treatment for antihistamine-refractory chronic urticaria. However, post-injection urticaria exacerbation and related adverse reactions remain inconsistently reported. This scoping review mapped evidence on clinical presentation, timing, mechanisms, management strategies, rechallenge outcomes, treatment discontinuation, and patient outcomes associated with these reactions. Methods: The review followed the Joanna Briggs Institute methodology and was registered prospectively in INPLASY (INPLASY202650060). PubMed, Scopus, and the Directory of Open Access Journals were searched from inception to April 2026. Eligible studies included English-language case reports, case series, observational studies, cohort studies, registry or pharmacovigilance studies, postmarketing surveillance studies, and clinical trials reporting urticaria-related or hypersensitivity-type adverse reactions during omalizumab treatment for chronic urticaria. Data were charted descriptively and synthesized narratively. Results: Seventeen studies published between 2015 and 2026 included 2790 patients with chronic urticaria, 60 of whom experienced urticaria-related adverse reactions, corresponding to a mapped proportion of 2.2%. Clinical phenotypes included transient urticaria exacerbation, urticaria flare or worsening, localized urticarial plaques at the injection area, angioedema, anaphylaxis or anaphylaxis-like reactions, and serum sickness-like reactions. Reactions occurred within minutes to several hours, within 24 h, several days, or up to 1 week after injection or dose escalation. Reactions occurred following the first dose, repeated doses, and dose escalation. Proposed mechanisms included delayed hypersensitivity, possible IgE-mediated delayed-onset anaphylaxis, serum sickness-like reaction, paradoxical worsening, infection-associated flare, excipient-related reactions, and severe underlying CSU exacerbation mimicking anaphylaxis. Management strategies ranged from observation or symptomatic treatment to discontinuation of omalizumab and systemic interventions. Conclusions: Omalizumab-associated urticaria-related adverse reactions were infrequently reported across the mapped evidence but were clinically heterogeneous. Standardized definitions and detailed case reporting are needed to clarify causality, recurrence risk, rechallenge safety, and long-term outcomes.

## 1. Introduction

Chronic urticaria is a common dermatologic disorder characterized by recurrent transient wheals, angioedema, or both, persisting for more than 6 weeks and frequently lasting for several years [1]. It is broadly classified into chronic spontaneous urticaria (CSU), in which symptoms occur in the absence of an identifiable trigger; and chronic inducible urticaria (CIndU), in which symptoms are reproducibly elicited by specific external stimuli, including cold, heat, pressure, friction, water, vibration, sweating, exercise, sunlight, ultraviolet radiation, and contact with certain agents [1]. These two forms may coexist within the same patient. Chronic urticaria represents a substantial public health burden. Between 1990 and 2021, the estimated number of prevalent cases worldwide increased from approximately 47.9 million to 66.5 million, while incident cases increased from 84.9 million to 117 million, although age-standardized prevalence and incidence rates remained relatively stable over time [2]. Disease burden is greater among females, children, and populations in low- to middle-Socio-demographic Index (SDI) regions, with considerable geographic variation across countries. The global lifetime prevalence of chronic urticaria has been estimated at 4.4%, with a point prevalence of approximately 0.7%, and women account for up to 80% of affected individuals [3]. Angioedema occurs in approximately 40–60% of patients, while concomitant CIndU has been reported in 7–30% of adults with CSU. In adults, the mean duration of CSU has been estimated at 11.5 ± 10.8 years, and spontaneous remission within 5 years occurs in only 30–55% of patients [3].

Management strategies differ according to disease subtype and level of disease control. In patients with CIndU, avoidance of identified triggers constitutes a key component of management and is typically combined with symptomatic pharmacological therapy [4]. CSU is primarily managed pharmacologically, beginning with standard-dose non-sedating or minimally sedating second-generation H1 antihistamines, followed by dose escalation up to fourfold in patients with inadequate symptom control. For patients who remain uncontrolled despite up-dosed antihistamines, add-on omalizumab is recommended. Escalation of therapy is clinically important because approximately 50% of patients do not respond adequately to standard-dose H1 antihistamines, nearly 60% continue to exhibit disease activity despite licensed-dose antihistamines, and up to 25% ultimately require third- or fourth-line therapies, including omalizumab or ciclosporin [3,5,6].

Omalizumab is a recombinant humanized monoclonal anti-immunoglobulin E (IgE) antibody that selectively binds to the Cε3 domain of IgE, which mediates IgE interaction with the high-affinity IgE receptor (FcεRI) [7]. By forming complexes with circulating free IgE, omalizumab prevents IgE binding to effector cells, including mast cells and basophils, thereby reducing cellular activation and inflammatory mediator release. This mechanism results in rapid, dose-dependent suppression of serum-free IgE concentrations. Omalizumab has demonstrated significant efficacy in chronic urticaria, particularly at a dose of 300 mg administered every 4 weeks. In a systematic review of 10 clinical trials involving 1620 patients, omalizumab 300 mg reduced the Urticaria Activity Score over 7 days (UAS7) by 11.05 points and significantly increased complete response rates compared with standard care (43% vs. 8.6%; relative risk [RR], 4.12) [8]. Another meta-analysis demonstrated significant improvements in weekly itch score, weekly hive score, UAS7, and Dermatology Life Quality Index (DLQI) compared with placebo [9].

The safety profile of omalizumab in CSU is generally favorable, with randomized trials and pooled analyses demonstrating no significant differences from placebo in overall adverse events, serious adverse events, or severe adverse events [9,10]. Nevertheless, post-injection reactions remain clinically important. Anaphylaxis is rare, with reported incidences of approximately 0.1% in premarketing clinical trials, 0.2% in postmarketing surveillance, and 0.09% in a Joint Task Force review [10]. Additionally, urticaria flares and other urticaria-related reactions following omalizumab administration have been reported in both case-based and real-world evidence. Ertaş et al. reported four patients with severe antihistamine-resistant CSU who developed angioedema, anaphylaxis, or urticaria flare-ups following omalizumab administration and noted that similar reactions had not been previously reported outside phase studies [11]. Subsequent observational evidence also documented additional urticaria-related adverse events, including urticaria reported as an adverse event in 5 of 280 patients (1.8%) and as a serious adverse event in 1 patient (0.4%) in a Japanese postmarketing surveillance study [12], transient urticaria exacerbation in 3 of 82 patients (3.7%) in a real-world study [13], and angioedema or urticaria flare-up in 31 of 1859 patients (1.7%) in a multinational real-world cohort [14].

Despite these reports, evidence regarding omalizumab-associated post-injection urticaria exacerbation and urticaria-related adverse reactions remains fragmented. Reported events have been described across case reports, observational studies, postmarketing surveillance studies, and real-world cohorts. However, definitions, timing, clinical characteristics, causal interpretations, management strategies, rechallenge outcomes, and treatment-discontinuation data are inconsistently reported. Consequently, uncertainty remains regarding the differentiation of post-injection urticaria exacerbation from baseline CSU activity, disease flare, anaphylaxis or anaphylaxis-like reactions, serum sickness-like reactions, and other hypersensitivity-type adverse reactions. Therefore, this scoping review aimed to map the available evidence regarding omalizumab-associated post-injection urticaria exacerbation and urticaria-related adverse reactions in patients with CSU or chronic urticaria. Specifically, this review summarized reported clinical presentations, timing of onset following omalizumab administration, proposed mechanisms, management strategies, treatment discontinuation or rechallenge, and subsequent clinical outcomes. This synthesis was intended to clarify the current state of evidence and identify knowledge gaps requiring further investigation.

## 2. Materials and Methods

### 2.1. Study Design and Protocol Registration

This scoping review was conducted in accordance with the Joanna Briggs Institute methodology for scoping reviews [15]. An a priori protocol was developed and prospectively registered with the International Platform of Registered Systematic Review and Meta-analysis Protocols (INPLASY202650060; DOI: 10.37766/inplasy2026.5.0060) on 10 May 2026, before the literature search, study selection, data extraction, and evidence synthesis were conducted. The initial database search was conducted on 11 May 2026. Screening, data extraction, evidence charting, narrative synthesis, and manuscript preparation were conducted between 11 May and 4 June 2026. The final search update was conducted on 31 May 2026 before manuscript finalization.

This review aimed to map the available evidence regarding post-injection urticaria exacerbation and urticaria-related adverse reactions occurring during omalizumab treatment for chronic urticaria. Specifically, the review sought to summarize the reported clinical presentations, timing of onset following omalizumab injection, proposed mechanisms, management strategies, and subsequent patient outcomes. The review question was as follows: “What evidence is available regarding urticaria exacerbation and urticaria-related adverse reactions following omalizumab treatment for chronic urticaria, including their clinical presentation, timing, possible mechanisms, management, and outcomes?”

The target population consisted of patients with CSU or chronic urticaria receiving omalizumab therapy. The concept of interest was post-injection urticaria exacerbation and urticaria-related adverse reactions associated with the clinical use of omalizumab in any healthcare setting without geographic restriction.

### 2.2. Eligibility Criteria

The eligibility criteria were structured according to the population–concept–context (PCC) framework. The population included patients with CSU or chronic urticaria receiving omalizumab therapy. The concept of interest comprised post-injection urticaria exacerbation and urticaria-related adverse reactions following omalizumab administration, including clinical presentations, timing of symptom onset, proposed mechanisms, management strategies, and subsequent treatment outcomes. The context encompassed the clinical use of omalizumab in any healthcare setting, including dermatology, allergy/immunology, outpatient, hospital-based, and real-world practice settings, without geographic restriction.

Eligible sources of evidence included case reports, case series, observational studies, cohort studies, retrospective studies, prospective studies, registry studies, pharmacovigilance surveillance studies, and clinical trials reporting urticaria-related or hypersensitivity-type adverse reactions during omalizumab treatment for chronic urticaria. Only English-language studies published or indexed from database inception to 30 April 2026 were included, consistent with the registered protocol.

### 2.3. Search Strategy

The search strategy was developed to identify published studies reporting omalizumab-associated post-injection urticaria exacerbation and urticaria-related adverse reactions during treatment for chronic urticaria. A three-step search strategy was employed. First, an initial limited search of PubMed and Scopus was conducted to identify relevant articles. Text words contained in the titles and abstracts of relevant articles, together with relevant index terms, were then used to develop the full search strategy.

The final search strategy was adapted for each database and information source. The databases searched included PubMed, Scopus, and the Directory of Open Access Journals (DOAJ). Searches were conducted from database inception to the pre-specified eligibility cut-off of 30 April 2026 and were limited to English-language publications. The initial database search was conducted on 11 May 2026, after INPLASY registration. The final search update was conducted on 31 May 2026 to confirm that all additional eligible studies published or indexed within the predefined eligibility period had been included. Therefore, May 2026 refers to the date on which the search was run or updated, whereas 30 April 2026 represents the pre-specified publication/indexing cut-off for eligible records. Additionally, the reference lists of included studies and relevant review articles were screened to identify additional eligible studies. No geographic restrictions were applied. Database-specific filters were used, where available, to limit results to relevant publication types, including original articles, letters, and notes in Scopus. The search was designed to capture case reports, case series, observational studies, cohort studies, retrospective and prospective studies, registry studies, pharmacovigilance surveillance studies, and clinical trials reporting relevant adverse events.

The Scopus search strategy was as follows:

TITLE-ABS-KEY ((omalizumab OR xolair OR “anti-IgE” OR “anti IgE” OR “anti-immunoglobulin E” OR “anti immunoglobulin E”) AND (“chronic spontaneous urticaria” OR “chronic idiopathic urticaria” OR “chronic urticaria” OR CSU OR CIU) AND (exacerbation OR flare OR flares OR worsening OR aggravation OR “urticaria flare” OR “urticarial flare” OR “disease flare” OR “disease exacerbation” OR “post-injection” OR “post injection” OR “after injection” OR “following injection” OR “delayed reaction” OR “delayed hypersensitivity” OR “paradoxical reaction” OR “paradoxical exacerbation” OR “injection reaction” OR “injection-site reaction” OR “adverse event” OR “adverse reaction” OR “adverse drug reaction” OR “treatment-emergent”)) AND (PUBYEAR < 2027) AND (LIMIT-TO (DOCTYPE, “ar”) OR LIMIT-TO (DOCTYPE, “le”) OR LIMIT-TO (DOCTYPE, “no”)) AND (LIMIT-TO (LANGUAGE, “English”))

This strategy was adapted for PubMed and DOAJ using database-specific syntax, field tags, and available filters.

During revision, and in response to peer-review feedback, a supplementary sensitivity search was performed in PubMed, Scopus, and DOAJ using additional phenotype-specific adverse-reaction terms, including “angioedema,” “anaphylaxis,” “hypersensitivity,” “injection-site urticaria,” “injection site urticaria,” “delayed anaphylaxis,” “excipient reaction,” “excipient hypersensitivity,” “adverse effect,” “side effect,” “serum sickness,” and “serum sickness-like reaction.” This supplementary search was conducted to assess whether the original registered search strategy had missed eligible sources using more specific adverse-reaction terminology. Newly identified records were screened using the same predefined eligibility criteria. The full original database search strategies and the supplementary sensitivity search strategies performed during revision are provided in Supplementary Table S2.

### 2.4. Study and Source of Evidence Selection

Following the search, all identified citations were collated and imported into EndNote X9 (Clarivate Analytics, Philadelphia, PA, USA), and duplicate records were removed. After a pilot screening process, two reviewers independently screened titles and abstracts against the predefined eligibility criteria. Sources considered potentially relevant were retrieved in full text and assessed further for eligibility.

The full texts of selected citations were independently reviewed by two reviewers according to the PCC criteria. Reasons for exclusion at the full-text stage were recorded and reported in the review. Disagreements during title/abstract screening or full-text assessment were resolved through discussion, and, when necessary, consultation with a third reviewer.

The search and study-selection process was reported in full and presented using a PRISMA flow diagram. The source-selection process followed the principles of transparent reporting for scoping reviews and was guided by the PRISMA Extension for Scoping Reviews and the PRISMA 2020 statement [16,17].

### 2.5. Data Extraction

Data were extracted independently by two reviewers using a standardized data-charting form developed specifically for this scoping review. The draft data-charting form, including all prespecified extraction domains, is provided in Supplementary Table S1. The form was designed to capture information relevant to the review question and the PCC framework.

Extracted data included author name, year of publication, country, study design, source type, participant characteristics, type of chronic urticaria, omalizumab dose and treatment schedule, duration of omalizumab treatment before the adverse event, type of urticaria-related or hypersensitivity-type adverse reaction, timing of onset after injection, clinical presentation, proposed mechanism, management strategies, treatment discontinuation or rechallenge, subsequent treatment, clinical outcome, and key conclusions reported by the study authors.

The draft data-charting form was piloted using a sample of included studies and refined as necessary to improve clarity and consistency. Any modifications to the extraction form were documented during the review process. Disagreements between reviewers were resolved through discussion and, when necessary, consultation with a third reviewer. Where relevant information was missing or unclear, study authors were contacted, where appropriate, to request additional data.

Critical appraisal of individual sources of evidence was not performed because the purpose of this scoping review was to map the extent, range, and characteristics of the available evidence rather than to assess the certainty of effect estimates or exclude studies based on methodological quality.

### 2.6. Data Analysis and Presentation

Extracted data were analyzed descriptively and mapped according to the review objective and research question. Study characteristics and key findings were summarized in tabular form, including author and year, country, study design, population, omalizumab treatment details, type of urticaria-related or hypersensitivity-type adverse reaction, timing of onset following injection, clinical presentation, management strategies, and patient outcomes.

The evidence was synthesized narratively according to the main concepts of interest, namely clinical presentation, timing after omalizumab administration, proposed mechanisms, management strategies, treatment discontinuation or rechallenge, and subsequent outcomes. Where appropriate, findings were grouped according to source type, including case reports or case series, observational studies, real-world studies, registry studies, pharmacovigilance or database studies, and clinical trials.

The results were presented using descriptive tables and a PRISMA flow diagram. A narrative summary accompanied the tabulated findings to explain how the evidence addressed the review question and to identify patterns, evidence gaps, and areas requiring further investigation. Meta-analysis was not performed because the objective of this scoping review was to map the available evidence, and substantial heterogeneity was anticipated across study designs, populations, adverse-event definitions, and outcome reporting methods.

For case-based reports with sufficient patient-level information, a structured causality-plausibility assessment was performed descriptively. This assessment was informed by the World Health Organization–Uppsala Monitoring Centre (WHO-UMC) causality assessment system and considered the temporal relationship, clinical phenotype compatibility, dechallenge, rechallenge or recurrence after re-exposure, objective or laboratory support, alternative explanations, and overall causality plausibility [18]. Formal numerical causality scoring was not applied because the included reports varied in completeness and often lacked sufficient information for standardized scoring across all domains. The structured causality-plausibility assessment for case-based reports is provided in Supplementary Table S3.

## 3. Results

**Figure 1:**
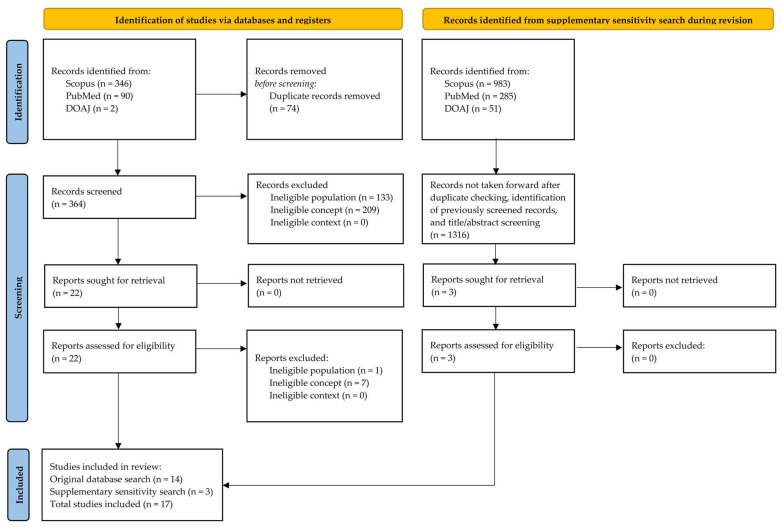
PRISMA 2020 flow diagram. The original database search followed the prospectively registered protocol. During revision, a supplementary sensitivity search was performed in PubMed, Scopus, and DOAJ using additional phenotype-specific adverse-reaction terms. Three additional eligible case-based reports were identified and incorporated into the revised synthesis. DOAJ, Directory of Open Access Journals.

The database search identified 438 records. After removal of duplicates and title/abstract screening, 22 reports were assessed for full-text eligibility. During revision, a supplementary sensitivity search using additional phenotype-specific adverse-reaction terms identified three further eligible case-based reports. Therefore, 17 studies were included in the final revised synthesis. The study-selection process, including reasons for exclusion, is summarized in Figure 1.

The 17 included studies were published between 2015 and 2026 and comprised 8 case reports, case series, letters, or correspondence reports [11,19,20,21,22,23,24,25], 6 retrospective studies, cohort studies, or chart reviews [13,14,26,27,28,29], and 3 prospective observational, postmarketing surveillance, or real-world studies [12,30,31]. The included evidence was geographically diverse, with studies conducted in Italy, Belgium, China, Greece, Israel/Georgia, Japan, Turkey, the United Kingdom, and the United States, as well as one multinational cohort.

Most studies focused on adult populations [11,13,19,21,22,23,24,25,26,27,28,29], whereas three studies involved pediatric populations [20,30,31], and two studies included mixed-age populations [12,14]. Across the included studies, the total chronic urticaria population comprised 2790 patients, among whom 60 patients were reported to have urticaria-related adverse reactions. However, these figures should be interpreted cautiously because several studies reported adverse events at the study level rather than at the individual-patient level, and some denominators were not fully stratified for the chronic urticaria subgroup.

The most commonly reported omalizumab regimen was 300 mg administered every 4 weeks or monthly [11,12,13,14,20,21,22,24,26,31]. A minority of studies described treatment initiation at 150 mg, dose escalation from 150 mg to 300 mg, or dose escalation above the standard 300 mg dose [14,22,25,30]. In some studies, omalizumab dose and schedule were not reported in the extracted data [27,28,29].

The level of clinical detail varied substantially according to study design. Case reports, case series, letters, and correspondence reports provided detailed descriptions of timing after injection, clinical presentation, suspected mechanisms, management, and outcomes [11,19,20,21,22,23,24,25]. In contrast, larger observational, postmarketing, real-world, and cohort studies primarily reported adverse events at the aggregate level, with limited information regarding patient-level timing, causal assessment, management decisions, rechallenge, and long-term outcomes [12,13,14,26,27,28,29,30,31]. The characteristics of the included studies and patient populations are summarized in Table 1.

Across the 17 included studies, omalizumab-associated urticaria-related reactions were reported as urticaria flare or worsening, angioedema, anaphylaxis or anaphylaxis-like reactions, serum sickness-like reactions, transient urticaria exacerbation, and localized urticarial plaques at the injection area. Detailed patient-level clinical information was available in 8 case reports, case series, letters, or correspondence reports, whereas the remaining 9 cohort, surveillance, real-world, or observational studies primarily reported adverse events at the aggregate level without detailed descriptions of timing or symptom patterns for individual affected patients [12,13,14,26,27,28,29,30,31].

Among the 60 affected patients identified across the included studies, 49 patients were reported in larger observational, real-world, cohort, or surveillance studies. These included 31 of 1859 patients (1.7%) in the multinational cohort, 5 of 280 patients (1.8%) in the Japanese postmarketing surveillance study, 4 of 9 CSU patients in the pediatric observational study, 3 of 82 patients (3.7%) in one retrospective cohort, 2 of 46 patients (4.3%) in the UK retrospective case-note review, 1 of 235 patients (0.4%) in the Belgian chart review, 1 of 141 CSU patients (0.7%) in the Turkish tertiary referral allergy-center experience, 1 of 61 pediatric patients (1.6%) in the multicenter pediatric real-world study, and 1 of 24 patients (4.2%) in the treatment-resistant CSU cohort [12,13,14,26,27,28,29,30,31]. The remaining 11 affected patients were reported in case reports, case series, letters, or correspondence reports with more detailed patient-level clinical descriptions [11,19,20,21,22,23,24,25].

The timing of onset varied considerably among reports with available data. Reactions occurred within a few minutes after the first injection, with recurrent anaphylactic episodes at 24 and 36 h [21]; within 30 min to more than 12 h after injection [11]; approximately 12–24 h after injection [19]; within 24 h after the first dose [30]; 1 day after the second dose [27]; 2.5 h or on the day of dosing [28]; 2 days after the first injection and after subsequent injections [24]; several days after dose escalation [25]; a few days after first administration [23]; 1 week after the second injection [20]; and 1 week after dose escalation [22]. Reactions occurred after the first administration, after repeated doses, after rechallenge, and after dose escalation, indicating that onset was not limited to initial exposure [11,19,20,21,22,23,24,25,27,28,30].

The severity of reactions ranged from transient or mild urticaria exacerbation to severe systemic reactions requiring treatment discontinuation, hospitalization, epinephrine administration, systemic corticosteroids, ciclosporin, oxygen, or other interventions [11,13,19,20,21,23,24,26,27,28]. Recurrence following subsequent omalizumab administration was clearly described in selected reports in which treatment was continued, repeated, or rechallenged after an initial reaction [11,19,20,24,28]. The clinical presentation, timing, severity, duration, and recurrence of reported reactions are summarized in Table 2.

Proposed mechanisms were described in greater detail in the case-based studies than in the larger observational studies. Among the 17 included studies, 8 case reports, case series, letters, or correspondence reports discussed possible mechanisms or differential explanations, whereas the remaining 9 cohort, surveillance, real-world, or observational studies provided limited or no patient-level mechanistic interpretation for urticaria-related adverse events [12,13,14,26,27,28,29,30,31].

The reported mechanisms were heterogeneous and included delayed hypersensitivity, possible IgE-mediated delayed-onset anaphylaxis, triphasic anaphylaxis, serum sickness-like reactions, nonhistaminergic or possibly bradykinin-mediated angioedema, paradoxical CSU worsening, infection-associated flare, possible excipient-related reactions, nocebo effect or overlap between side effects and uncontrolled disease activity, and severe CSU exacerbation mimicking anaphylaxis rather than true omalizumab-induced anaphylaxis [11,14,19,20,21,22,23,24,25,28]. Evidence supporting an association with omalizumab included temporal onset after injection, recurrence following continued dosing or rechallenge, improvement after discontinuation, positive intradermal testing, laboratory abnormalities, multisystem involvement compatible with anaphylaxis, response to epinephrine or targeted treatment, and prevention of recurrence with adjunctive therapy, depending on the study [11,19,20,21,22,23,24,25].

Alternative explanations were variably considered and included spontaneous CSU exacerbation, uncontrolled baseline disease, concomitant or external triggers, infection, autoimmune disease activity, malignancy, parasitic infestation, hormonal sensitivity, histaminergic or complement-mediated angioedema, and omalizumab-induced anaphylaxis as a differential diagnosis in anaphylaxis-like presentations [11,19,20,22,23,24,25,28]. Reported or suspected predisposing factors included severe antihistamine-resistant CSU, baseline angioedema, dose escalation, repeated exposure after an initial reaction, possible subclinical dysimmune status, parasite exposure in endemic settings, complex or severe CSU, previous anaphylaxis-like episodes before omalizumab, female sex, chronic inducible urticaria, worse baseline disease control, and fast response failure [11,14,20,21,22,23,24,25,28]. The proposed mechanisms, differential considerations, risk factors, and causality interpretations are summarized in Table 3. Because patient-level causality data were most complete in the case-based reports, a structured WHO-UMC-informed causality-plausibility assessment was performed for these reports. Overall causality plausibility was classified as probable/likely in several reports with compatible timing, clinical phenotype, dechallenge, recurrence, rechallenge, or objective support; possible in heterogeneous cases where spontaneous CSU fluctuation or other explanations remained plausible; and unlikely for direct omalizumab hypersensitivity when an alternative cause was more strongly supported. This assessment is summarized in Supplementary Table S3.

Management strategies were most comprehensively described in the 8 case reports, case series, letters, or correspondence reports and in selected retrospective reports with available clinical details. Reported interventions included antihistamines, systemic corticosteroids, intravenous fluids, epinephrine, oxygen, ciclosporin, tranexamic acid, and treatment of alternative or contributing conditions, such as parasitic infection or suspected hormonal contribution to recurrent urticaria/angioedema [11,19,20,21,22,23,24,25,27,28]. In contrast, patient-level management details were limited or unavailable in several larger cohort, surveillance, real-world, and observational studies [12,13,14,26,29,30,31].

Permanent discontinuation, temporary discontinuation, or no further omalizumab administration after the reaction was reported in selected studies, including 4 affected patients in one case series [11] and individual patients in several additional case-based or retrospective reports [19,20,21,22,23,24,25,27]. In contrast, treatment discontinuation was not required in cases involving transient or mild urticaria events or reactions judged most likely to reflect underlying CSU activity rather than true omalizumab-induced anaphylaxis [13,28,29,30,31]. In the larger observational studies, it could not always be clearly determined whether treatment discontinuation was specifically attributable to urticaria or angioedema flare-up [12,14,26].

Clinical outcomes ranged from complete resolution within minutes to days to persistent symptoms lasting several weeks or months [11,19,20,21,23,24,25,28]. Rechallenge, continuation, or repeated administration after the initial reaction was described in selected reports and was associated with recurrent or more severe reactions in some cases, whereas other patients continued treatment without recurrent complications [11,19,20,24,28,30]. Evidence regarding subsequent biologic therapy or cross-reactivity with other biologic agents was not consistently reported across the included studies. Management strategies, treatment decisions, clinical outcomes, and evidence gaps are summarized in Table 4.

## 4. Discussion

### 4.1. Principal Findings and Overall Evidence Map

**Figure 2:**
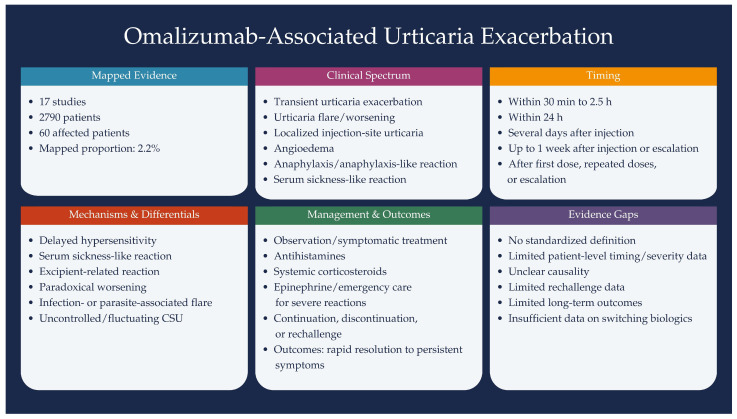
Evidence map of omalizumab-associated urticaria exacerbation and urticaria-related reactions in chronic urticaria. The figure summarizes the mapped evidence across 17 included studies, including the mapped patient count, clinical spectrum, timing after injection or dose escalation, possible mechanisms and differential explanations, management approaches, outcomes, and key evidence gaps. The mapped proportion should not be interpreted as an incidence estimate because the included studies were heterogeneous, and several larger studies reported adverse events at the aggregate level rather than at the affected-patient level.

This scoping review mapped 17 studies published between 2015 and 2026 describing omalizumab-associated post-injection urticaria exacerbation and urticaria-related adverse reactions in patients with chronic urticaria. Across the included evidence, 60 affected patients were identified among 2790 patients, corresponding to a mapped proportion of 2.2%. This figure should not be interpreted as an incidence estimate because the included studies differed in design, denominator reporting, adverse-event definitions, and level of patient-level detail. Most affected patients were identified in larger real-world, cohort, or postmarketing studies, whereas case reports, case series, letters, and correspondence reports provided the most detailed descriptions of timing, clinical presentation, management, rechallenge, and outcomes. The clinical spectrum was broad, ranging from mild transient urticaria exacerbation to urticaria flare or worsening, localized urticarial plaques at the injection area, angioedema, anaphylaxis or anaphylaxis-like reactions, and serum sickness-like reactions. Figure 2 provides an overall visual summary of the mapped evidence, including the mapped patient count, mapped proportion, clinical spectrum, timing, proposed mechanisms, management strategies, and major evidence gaps associated with omalizumab-associated urticaria-related reactions.

The findings of this scoping review should be interpreted within the broader context of biologic-associated hypersensitivity. Urticaria-related reactions, angioedema, anaphylaxis, delayed localized injection-site urticaria, serum sickness-like reactions, and other hypersensitivity phenotypes have been described across multiple biologic classes [32,33]. Reslizumab carries an anaphylaxis risk of approximately 0.1–1%, with reactions typically occurring during or within 20 min of infusion, whereas benralizumab-associated hypersensitivity reactions, including urticaria, angioedema, and anaphylaxis, occur in approximately 3% of patients [32]. Mepolizumab is primarily associated with injection-site reactions, reported in approximately 3–9% of patients, with no reported cases of anaphylaxis. These comparisons suggest that urticaria-related and systemic hypersensitivity reactions are not unique to omalizumab but instead represent part of a broader spectrum of biologic-associated adverse reactions.

Another biologic agent used in chronic urticaria is dupilumab [4,34]. Dupilumab has been associated with hypersensitivity reactions in approximately 0.1–1% of patients, injection-site reactions in approximately 8–19%, and very rare serum sickness-like reactions occurring in less than 0.01% [32]. One case report further illustrates the heterogeneous nature of dupilumab-associated reactions, including diffuse pruritic wheals occurring approximately 12 h after each injection and controlled with antihistamine prophylaxis [35], delayed localized injection-site urticaria after repeated doses managed with topical corticosteroids without discontinuation [36], and severe progressive angioedema with episodic urticaria requiring treatment discontinuation [37]. Newer therapies for chronic urticaria also demonstrate distinct safety profiles relevant to the interpretation of omalizumab-associated reactions. Ligelizumab, a high-affinity anti-IgE antibody, has been associated with a higher incidence of mild-to-moderate injection-site reactions and erythema compared with both omalizumab and placebo, although anaphylaxis has not been reported [33]. Remibrutinib, an oral Bruton tyrosine kinase inhibitor, provides a different safety comparison because it targets intracellular signaling involved in mast cell and basophil activation [38]. Although overall adverse events, serious adverse events, and treatment discontinuation due to adverse events were not significantly different from placebo, remibrutinib was associated with increased risks of total infections (RR, 1.59), upper respiratory tract infections (RR, 2.89), and petechiae (RR, 7.53). Long-term extension data also identified infections, bleeding, and cytopenias as adverse events of special interest, occurring in 23%, 4.4%, and 0.5% of patients, respectively [33]. Notably, urticaria-related adverse reactions have not been reported with remibrutinib.

### 4.2. Clinical Heterogeneity and Timing of Omalizumab-Associated Urticaria-Related Reactions

The timing and clinical phenotype of omalizumab-associated urticaria-related reactions were heterogeneous across the included studies. Reported onset ranged from within minutes to several hours after injection, within 24 h, approximately 12–24 h, several days, or up to 1 week after injection or dose escalation. Reactions occurred after first administration, repeated doses, and dose escalation, indicating that post-injection monitoring should not be restricted to the initial exposure. Importantly, post-injection worsening should not be interpreted as a single uniform clinical entity. The reported phenotypes included transient CSU flare, urticaria worsening, localized urticarial plaques at the injection area, angioedema, anaphylaxis or anaphylaxis-like reactions, and serum sickness-like reactions. This distinction is clinically important because these entities differ in timing, severity, recurrence risk, causality interpretation, and management strategies. Therefore, careful assessment of symptom onset, lesion morphology, systemic manifestations, recurrence following subsequent injections, and alternative explanations is required before attributing post-injection urticaria worsening directly to omalizumab.

The broader biologic literature similarly supports the concept that urticaria-related and hypersensitivity-type reactions are clinically heterogeneous and vary substantially in timing. Immediate reactions have been described with rituximab and infliximab, typically occurring during infusion or within the first hour, whereas delayed reactions may occur hours to weeks after administration, including immune checkpoint inhibitor-associated urticaria developing within 6 weeks of treatment initiation and delayed serum sickness-like reactions reported with other biologics [39,40,41]. Clinical manifestations also vary across biologic agents, ranging from isolated pruritic wheals after immune checkpoint inhibitors to localized injection-site reactions after adalimumab, recurrent urticaria after infliximab, angioedema or anaphylaxis after rituximab, and severe first-dose anaphylaxis after cetuximab [39,40,41,42]. These reactions are not limited to initial exposure. Immune checkpoint inhibitor-associated urticaria has been reported after the first or second treatment cycle, rituximab reactions after the first or second dose, infliximab reactions after both early and later infusions, and adalimumab injection-site reactions after repeated dosing [39,40,42]. Mechanistically, biologic-associated reactions may involve distinct and potentially overlapping pathways, including IgE-mediated or non-IgE-mediated reactions, cytokine-release reactions, delayed cellular hypersensitivity, serum sickness-like reactions, and pre-existing cross-reactive IgE, as described for cetuximab and alpha-gal sensitization [41,42]. Recognition of the diversity of hypersensitivity reactions across biologic therapies highlights the importance of individualized monitoring strategies and standardized diagnostic criteria in clinical practice. Future systematic data collection and mechanistic studies are needed to improve patient selection, optimize management strategies, and minimize adverse outcomes associated with biologic therapies.

### 4.3. Mechanistic Interpretations and Challenges in Causal Attribution

When urticaria-related reactions occur during omalizumab treatment, a broad differential diagnosis should be considered before concluding that the event represents a direct omalizumab-associated adverse reaction. Post-injection worsening may reflect several distinct entities, including background CSU activity, spontaneous disease fluctuation, uncontrolled CSU, inadequate background treatment, inducible urticaria or trigger exposure, omalizumab-related immediate or delayed hypersensitivity, anaphylaxis or anaphylaxis-like reactions, serum sickness-like reactions, paradoxical worsening, excipient-related hypersensitivity, infection-associated flare, parasitic infestation, autoimmune or autoinflammatory disease activity, concomitant medication-induced urticaria or angioedema, idiopathic anaphylaxis, or other mast-cell-mediated disorders.

Background chronic urticaria activity represents an important differential diagnosis when symptoms worsen during omalizumab therapy. Chronic urticaria is inherently fluctuating, and symptoms may worsen because of spontaneous disease activity, uncontrolled baseline inflammation, inadequate background treatment, or exposure to inducible or aggravating triggers. Previous chronic urticaria literature emphasizes that outbreaks or swelling episodes may be triggered or aggravated by stress, foods, nonsteroidal anti-inflammatory drugs (NSAIDs), trauma, infections, or other external stimuli [4,43,44]. This is particularly relevant because patients requiring omalizumab often have difficult-to-control disease, including antihistamine-refractory CSU or incomplete response to standard therapy [4,44,45]. Furthermore, CIndU may coexist with CSU, and trigger exposure may mimic treatment-related worsening; therefore, physical or environmental triggers should be assessed before attributing post-injection symptoms directly to omalizumab [4,46].

Formulation-related reactions should also be considered when urticaria-related symptoms occur after omalizumab administration, particularly when the clinical pattern suggests hypersensitivity but the active drug itself may not be the only plausible trigger. Omalizumab contains several non-medicinal ingredients, including L-arginine hydrochloride, L-histidine, L-histidine hydrochloride monohydrate, and polysorbate 20 [47]. Among these, polysorbates are clinically relevant because excipient-related hypersensitivity has been described with both biologic and non-biologic formulations. In the broader excipient literature, polysorbate 80 has been implicated in generalized urticaria and angioedema, supported by positive intradermal testing and subsequent tolerance of polysorbate-free formulations [48]. Polysorbate-containing preparations have also been associated with urticarial reactions that resolved after discontinuation and recurred following rechallenge, supporting a formulation-related mechanism in selected cases [49]. For omalizumab specifically, polysorbate 20 has been proposed as a potential contributor to hypersensitivity reactions, including anaphylaxis and serum sickness-like reactions, with reports of positive skin testing to polysorbate 20 in patients receiving Xolair^®^ [50]. Confirmation of excipient- or formulation-related hypersensitivity requires careful allergy evaluation, including skin prick testing with the suspected product and relevant excipients, stepwise intradermal testing when appropriate, patch testing for delayed reactions, and selected in vitro assays such as basophil activation testing, histamine-release assays, or anti-excipient IgE/IgG testing [51,52,53,54,55]. Controlled provocation or challenge testing may provide diagnostic confirmation, but should be reserved for carefully selected cases because of the risk of severe reactions, particularly when polyethylene glycol or polysorbate hypersensitivity is suspected [54,55,56]. Clinical tolerance of an alternative formulation lacking the suspected excipient may further support excipient causality [48]. Although excipient-related hypersensitivity appears uncommon and is not routinely evaluated in most omalizumab studies, it remains an important differential diagnosis when urticaria, angioedema, or anaphylaxis-like symptoms occur temporally after injection, particularly when reactions recur despite otherwise stable chronic urticaria control.

External or comorbid triggers should also be considered when urticaria-related symptoms develop during omalizumab treatment because infection, parasitic infestation, and autoimmune or autoinflammatory disease activity may independently aggravate urticaria. Infectious triggers have been associated with both acute and chronic urticaria, including viral infections, bacterial infections, Helicobacter pylori, urinary tract infection, streptococcal infection, and other inflammatory foci; evaluation generally includes clinical history, physical examination, complete blood count, inflammatory markers, cultures, serology, or targeted microbiological testing [57,58,59,60]. Parasitic infestation is another relevant consideration, particularly in endemic settings or in patients with gastrointestinal symptoms, eosinophilia, relevant travel or exposure history, or treatment-resistant urticaria. Reported investigations include stool examination, stool culture, parasite serology, specific IgE testing, and, in selected cases, histopathologic confirmation of the organism; improvement following antiparasitic therapy may further support causality [61,62,63,64,65]. Autoimmune activity may also contribute to chronic urticaria through IgG-mediated autoimmunity or IgE-mediated autoallergy, with reported associations involving thyroid autoimmunity, rheumatoid arthritis, systemic lupus erythematosus, celiac disease, and autoantibodies against FcεRI, IgE, or thyroid peroxidase. Evaluation may include antinuclear antibodies, rheumatoid factor, antithyroid antibodies, complement testing, autologous serum skin testing, basophil activation assays, or other immunologic investigations when clinically indicated [66,67,68]. Additionally, autoinflammatory disorders such as cryopyrin-associated periodic syndromes, Schnitzler syndrome, and tumor necrosis factor receptor-associated periodic syndrome may present with urticarial eruptions accompanied by systemic inflammatory features and may require evaluation of fever patterns, inflammatory markers, skin biopsy, and genetic testing in selected patients [69].

Medication-related and mast-cell-mediated mimickers should likewise be considered when urticaria, angioedema, or anaphylaxis-like symptoms occur during omalizumab therapy because these presentations may result from concomitant medications or underlying mast-cell disorders rather than omalizumab itself. Drug-induced urticaria or angioedema may be associated with NSAIDs, aspirin, antibiotics, opioids, radiocontrast agents, and angiotensin-converting enzyme (ACE) inhibitors. NSAIDs may exacerbate chronic urticaria through cyclooxygenase-1 inhibition, whereas ACE inhibitor-related angioedema is typically bradykinin-mediated and may occur without urticaria [70,71,72]. Medication causality assessment may involve a detailed medication timeline, evaluation of temporal relationships, dechallenge after withdrawal of the suspected medication, recurrence following inadvertent or supervised rechallenge, and selected allergy testing or drug-provocation testing when appropriate [72,73,74]. Idiopathic anaphylaxis and mast-cell activation disorders may also mimic biologic-associated hypersensitivity, particularly in patients presenting with recurrent anaphylaxis, urticaria, angioedema, hypotension, flushing, or respiratory symptoms without an identifiable trigger. Evaluation may include acute and baseline serum tryptase, urinary mast-cell mediators, flow cytometry for aberrant mast-cell markers, KIT D816V mutation testing, and bone marrow evaluation in selected patients with recurrent or severe episodes [75,76]. This distinction is clinically important because some patients initially diagnosed with idiopathic anaphylaxis may have an underlying clonal mast-cell disorder, and mast-cell activation syndrome requires both compatible clinical symptoms and objective evidence of mediator elevation with response to mast-cell-directed therapy [75,76].

Causality assessment was challenging because post-injection worsening may reflect either an omalizumab-associated adverse reaction or fluctuation of underlying CSU. Therefore, we used a structured WHO-UMC-informed causality-plausibility assessment for case-based reports with sufficient patient-level detail. Overall, causality appeared more plausible when reactions showed compatible timing, improvement after discontinuation, recurrence after repeated exposure or rechallenge, objective support, or careful exclusion of competing causes [19,20,21,23,24,25]. In contrast, causality remained less certain when spontaneous CSU fluctuation, treatment ineffectiveness, or alternative triggers such as parasitic infection remained plausible explanations [11,22]. These findings support standardized reporting of timing, baseline disease activity, dechallenge, rechallenge, objective investigations, and alternative explanations in future studies.

From a practical bedside perspective, several features may raise suspicion for an omalizumab-associated adverse drug reaction rather than spontaneous fluctuation of underlying CSU. These include onset within minutes to a few hours after injection; rapidly progressive generalized urticaria; angioedema; respiratory, cardiovascular, or gastrointestinal involvement; and reproducible recurrence after rechallenge or subsequent dosing [11,28,77,78]. Symptoms developing after 24 h may suggest delayed hypersensitivity or serum sickness-like reaction, particularly when accompanied by fever, arthralgia, malaise, lymphadenopathy, gastrointestinal symptoms, or low complement levels [19,23,25]. Localized urticaria confined to the injection area may indicate an injection-site reaction [29]. Therefore, clinical assessment should consider the time from injection, lesion distribution, systemic involvement, dose number or dose escalation, recurrence after re-exposure, response to dechallenge, and competing explanations such as infection, inducible triggers, concomitant medications, or uncontrolled baseline disease. No single feature confirms causality; however, systemic symptoms, recurrence after rechallenge, or improvement after discontinuation strengthen the plausibility of an omalizumab-associated adverse drug reaction [11,19,23,25,28,77,78].

### 4.4. Clinical Implications for Documentation, Monitoring, and Cautious Rechallenge

Across the 17 included studies, the mapped evidence indicates that clinical decision-making following omalizumab-associated urticaria-related reactions should be guided by timing, severity, recurrence, and diagnostic uncertainty rather than by temporal association alone. Reactions occurred after initial exposure, repeated doses, dose escalation, rechallenge, and subsequent injections. Previous safety reviews also reported that most omalizumab-associated anaphylaxis events occurred within the first three doses and within 1–2 h after administration, although delayed reactions occurring more than 12–24 h after injection have also been described [11,19,20,21,22,23,24,25,77,78]. Therefore, adverse-reaction monitoring should not be restricted to the first injection, and patients should receive education regarding delayed reactions and clear return precautions.

Management should be guided by reaction severity, ranging from continued treatment with observation for mild or transient urticaria exacerbation to withholding or discontinuing omalizumab and providing acute systemic treatment for severe, recurrent, or systemic reactions [11,13,19,20,21,23,24,27,28,29,30,31]. Careful documentation of symptom timing, clinical phenotype, systemic manifestations, dose context, baseline CSU activity, treatment response, and alternative explanations is essential before attributing post-injection worsening directly to omalizumab because excipient-related reactions, comorbid conditions, concomitant exposures, or underlying disease activity may confound causality assessment [12,14,19,20,22,24,28,78,79,80]. Rechallenge after a suspected adverse reaction should be individualized and approached cautiously, taking into account the severity of the initial event, anticipated treatment benefit, available alternatives, patient preference, and the feasibility of supervised management. Selected reports suggest that continuation, rechallenge, prophylaxis, or desensitization may be feasible in carefully selected patients; however, severe systemic reactions generally warrant avoidance of unsupervised re-exposure [24,28,30,77,81].

### 4.5. Evidence Gaps and Priorities for Future Research

This scoping review identified several important evidence gaps that should guide future research on omalizumab-associated post-injection urticaria exacerbation and urticaria-related adverse reactions. First, there is currently no standardized definition for post-injection urticaria exacerbation during omalizumab treatment. Across the included studies, events were variably described as urticaria flare-up, worsening of urticaria, transient exacerbation, angioedema, anaphylaxis or anaphylaxis-like reaction, and serum sickness-like reaction. This variability limits comparability across studies and complicates differentiation between true treatment-associated reactions and spontaneous CSU fluctuation, uncontrolled baseline disease, infection-associated flare, excipient-related hypersensitivity, or other mimicking conditions. Future studies should establish standardized criteria incorporating timing after administration, lesion morphology, presence or absence of angioedema, systemic manifestations, baseline CSU activity, recurrence after subsequent injections, and response to dechallenge or rechallenge.

Second, larger observational, postmarketing, and real-world studies provided useful estimates of event frequency but frequently lacked patient-level information regarding timing, clinical phenotype, management, causality assessment, and outcomes. In contrast, case reports and case series provided detailed clinical descriptions but were limited by small sample size and publication bias. This imbalance restricts the interpretation of the true clinical spectrum, recurrence risk, and prognostic significance of these reactions. Future prospective pharmacovigilance studies, registries, and multicenter cohort studies should use standardized adverse-event reporting forms capturing dose number, dose escalation, time from injection to onset, symptom duration, severity, treatment requirements, emergency visits or hospitalization, discontinuation, rechallenge, recurrence, and long-term disease control. These data would help clarify which reactions may be safely monitored, which require treatment discontinuation, and which warrant specialist allergy evaluation.

Third, evidence regarding diagnostic workup and mechanistic evaluation remains limited. Few studies reported objective biomarkers or structured investigations such as acute and baseline serum tryptase, complement levels, inflammatory markers, skin testing with omalizumab, excipient testing, basophil activation testing, anti-drug antibodies, anti-excipient antibodies, or formal causality assessment tools. Consequently, mechanistic interpretations often relied primarily on temporal association, recurrence, or clinical judgment. Future studies should incorporate structured diagnostic algorithms capable of distinguishing IgE-mediated reactions, non-IgE-mediated mast-cell activation, serum sickness-like reactions, excipient-related hypersensitivity, paradoxical CSU worsening, and unrelated disease flares. Additional research should also evaluate whether biomarkers, baseline CSU endotypes, autoimmune features, infection or parasite status, dose escalation, or prior mild post-injection symptoms predict recurrence or severe reactions.

Finally, future studies should address management questions directly relevant to clinical practice. These include whether omalizumab can be safely continued after a mild transient urticaria exacerbation, when rechallenge is appropriate, how rechallenge should be supervised, whether premedication or desensitization has a role, and which alternative therapies are safest after suspected omalizumab-associated urticaria-related reactions. Evidence regarding long-term outcomes after discontinuation, switching to another biologic, or using emerging therapies also remains limited. In the included studies, subsequent management after omalizumab discontinuation was limited to conventional therapies, including antihistamines, systemic corticosteroids, cyclosporine, or treatment of an alternative contributing condition, or was unreported. None of the included studies reported successful switching to dupilumab, ligelizumab, remibrutinib, or another biologic or targeted therapy without recurrence of similar reactions. Therefore, the safety and outcome of switching to alternative biologics or other targeted therapies after suspected omalizumab-associated urticaria-related reactions remains a critical knowledge gap. Addressing these gaps will require prospective patient-level data collection and harmonized reporting across dermatology, allergy, and pharmacovigilance settings. Such efforts would improve causal attribution, support risk-stratified management, and enhance clinician counseling after post-injection urticaria-related reactions.

### 4.6. Strengths and Limitations

This scoping review has several strengths. To our knowledge, it is among the first reviews specifically focused on mapping omalizumab-associated post-injection urticaria exacerbation and urticaria-related adverse reactions in patients with chronic urticaria. By focusing on this specific adverse-reaction pattern, the review addresses a clinically important topic that is often inconsistently reported within broader biologic safety studies. Another strength is the inclusion of both case-based reports and larger real-world, cohort, postmarketing, and retrospective studies. Case reports and case series provided detailed clinical information on timing, phenotype, management, rechallenge, and outcomes, whereas larger studies contributed broader safety data from routine clinical practice. Moreover, the review was guided by the PCC framework and conducted according to a prospectively registered protocol, thereby enhancing the transparency, structure, and reproducibility of the search strategy, study selection, data charting, and narrative synthesis.

Several limitations should also be acknowledged. First, although the electronic search followed the prospectively registered protocol and was supplemented during revision with additional phenotype-specific adverse-reaction terms, the information sources were restricted to PubMed, Scopus, and DOAJ. Additional bibliographic, regulatory, trial-registry, and pharmacovigilance sources, such as Embase, Web of Science, the Cochrane Library, ClinicalTrials.gov, WHO ICTRP, FDA FAERS, EMA/EudraVigilance, WHO VigiBase, manufacturer pharmacovigilance reports, and regulatory safety documents, were not systematically searched. Therefore, unpublished data, regulatory safety signals, spontaneous adverse-event reports, and non-indexed pharmacovigilance evidence may have been missed. Second, the search was restricted to English-language publications, which may have resulted in the exclusion of relevant studies published in other languages. Third, the included evidence was heterogeneous with respect to study design, population, clinical setting, omalizumab regimen, adverse-event terminology, and outcome reporting. This heterogeneity limited direct comparison across studies and precluded quantitative synthesis. Fourth, many larger observational, surveillance, real-world, and cohort studies reported adverse events only at the aggregate level, with limited patient-level information regarding timing, clinical morphology, systemic manifestations, management, rechallenge, discontinuation, and long-term outcomes. Fifth, causality could not be confirmed in many cases because objective investigations, structured causality assessment, dechallenge or rechallenge data, and evaluation of alternative explanations were inconsistently reported. Finally, although the review mapped the number of reported affected patients, it was not designed to estimate incidence or risk. The frequency of omalizumab-associated urticaria-related reactions cannot be reliably determined from scoping-review evidence because of differences in study design, denominators, adverse-event definitions, reporting thresholds, and the likely influence of publication and reporting bias.

## 5. Conclusions

This scoping review mapped the available evidence regarding omalizumab-associated post-injection urticaria exacerbation and urticaria-related adverse reactions in patients with chronic urticaria. Across 17 included studies, these reactions appeared uncommon but clinically heterogeneous, ranging from transient urticaria exacerbation to urticaria worsening, localized urticarial plaques at the injection area, angioedema, anaphylaxis or anaphylaxis-like reactions, and serum sickness-like reactions. Reaction onset varied from within minutes to several hours, within 24 h, several days, or up to 1 week after injection or dose escalation, and reactions occurred after initial exposure, repeated doses, rechallenge, or dose escalation.

These findings suggest that post-injection worsening should not be regarded as a single uniform event or attributed to omalizumab solely on the basis of temporal association. Clinical interpretation should consider symptom onset, clinical phenotype, systemic manifestations, baseline chronic urticaria activity, recurrence, response to dechallenge or rechallenge, and alternative explanations. Mild or transient reactions may be managed with observation and symptomatic treatment, whereas severe, recurrent, or systemic reactions require careful evaluation, possible treatment discontinuation, and appropriate acute management. Future studies should employ standardized definitions and detailed patient-level reporting of timing, severity, management, causality assessment, rechallenge outcomes, and long-term follow-up to support more accurate diagnosis and risk-stratified clinical management.

## Figures and Tables

**Table 1:** Characteristics of included studies and patient populations.

Author, Year	Reference	Country	Study Design/Source Type	Study Population	Sample Size	Type of Chronic Urticaria	Omalizumab Dose/Schedule	Duration of Treatment/Follow-Up	Study Aim/Purpose
Cekic, 2026	[30]	Turkey	Multicenter retrospective real-world study	Children and adolescents with CSU treated with omalizumab at five academic medical centers	61	CSU, with or without CIndU	150 mg every 4 weeks in 37 patients; 300 mg every 4 weeks in 24 patients; dose increased to 300 mg in selected patients initially receiving 150 mg	UAS7 assessed at baseline and at 1, 3, 6, and 12 months; relapse assessed within 6 months after omalizumab discontinuation	To investigate the real-life efficacy and safety of different omalizumab dosing regimens in pediatric CSU
Lestani, 2025	[23]	Italy	Case report	51-year-old woman with CSU	1	CSU	300 mg; schedule not reported	Reaction occurred a few days after the first administration	To report a case of serum sickness-like reaction after omalizumab administration and briefly review the literature
Soegiharto, 2025	[14]	Multinational	Retrospective multicenter cohort study	CU patients ever treated with omalizumab across 14 UCARE centers	1859	Mixed CU, including CSU, CIndU, or both	300 mg every 4 weeks in most patients; dose escalation above 300 mg in some patients	Median treatment duration was 17.0 months; maximum duration was 12.3 years	To investigate the spectrum and impact of reported side effects of omalizumab in a large, long-term, daily-practice CU cohort
Chen, 2024	[13]	China	Retrospective cohort study	Adult patients with antihistamine-refractory CSU	82	CSU	300 mg every 4 weeks	16 weeks	To examine the relationship between baseline biomarkers, clinical parameters, and clinical response to omalizumab
Konstantinou, 2024	[22]	Greece	Case report	28-year-old female agriculturist with antihistamine-refractory CSU	1	CSU	300 mg every 4 weeks, with dose escalation to 450 mg	Flare occurred 1 week after dose escalation, approximately 13–14 weeks after treatment initiation; total follow-up was 27 weeks	To present a case of paradoxical CSU exacerbation following omalizumab dose escalation, coinciding with parasitic infection
Galletta, 2023	[31]	Italy	Prospective observational study	Pediatric patients aged 6–18 years with severe asthma and/or CSU	23 total; 9 with CSU	CSU in the relevant subgroup; mixed asthma/CSU cohort overall	300 mg every 4 weeks for CSU	Median follow-up was 2 years, range 1–4 years	To evaluate the long-term real-life safety of omalizumab in pediatric patients with asthma and/or CSU
Hide, 2023	[12]	Japan	Observational postmarketing surveillance study	Japanese patients with CSU responding inadequately to conventional therapies	280	CSU	300 mg per administration in most patients; median interval was 30 days	52-week observation period; mean treatment duration was 195.6 days	To evaluate the longer-term safety and overall effectiveness of omalizumab in CSU in a real-world clinical setting
Öztop, 2022	[27]	Turkey	Retrospective real-world study/tertiary referral allergy-center experience	Patients treated with omalizumab across several indications, including a CSU subgroup	213 total; 141 with CSU	CSU in the relevant subgroup	Not reported in the extracted data	Ten-year center experience; individual treatment duration not reported in the extracted data	To evaluate long-term real-world omalizumab experience in a tertiary referral allergy center
Dies, 2020	[19]	Italy	Case report	33-year-old Caucasian woman with antihistamine-resistant CSU	1	CSU	300 mg; schedule not reported	Reaction occurred 12–24 h after the first and second doses	To report a severe adverse reaction to omalizumab therapy in CSU
Lapeere, 2020	[26]	Belgium	Retrospective chart review	Adults with CSU who initiated omalizumab treatment	235	CSU	300 mg in most patients; mean interval was 4.8 weeks	Mean omalizumab treatment duration was 11.9 months; mean observation period was 15.7 months	To collect real-world clinical data on omalizumab treatment in adults with CSU
Weiss, 2020	[25]	United States	Case report	28-year-old active-duty female with chronic idiopathic urticaria/angioedema	1	Chronic idiopathic urticaria	150 mg every 4 weeks, with dose escalation to 300 mg	Treated for 4 months at 150 mg; reaction occurred several days after the first 300 mg dose	To report a case of serum sickness-like reaction in an adult treated with omalizumab
Tabanlıoğlu-Onan, 2019	[29]	Turkey	Retrospective study	Patients with treatment-resistant CSU receiving omalizumab	24	CSU	Not reported in the extracted data	Not reported in the extracted data	To evaluate the efficacy and safety of omalizumab in treatment-resistant CSU
Eapen, 2018	[20]	United States	Case report/letter to the editor	Almost 12-year-old female with chronic idiopathic/spontaneous urticaria and angioedema	1	CIU/CSU with angioedema	300 mg monthly	Reaction developed 1 week after the second injection; symptoms worsened after each subsequent monthly injection over the next 6 months; evaluated 3 months after omalizumab discontinuation	To report a serum sickness-like reaction in a pediatric patient receiving omalizumab for chronic urticaria
Magen, 2018	[24]	Israel/Georgia	Case report/correspondence	26-year-old woman with severe antihistamine-resistant CSU without prior angioedema	1	CSU without baseline angioedema	300 mg monthly as add-on therapy to high-dose fexofenadine	Angioedema occurred 2 days after the first injection and recurred after subsequent injections; omalizumab was discontinued after 6 months and later restarted, with recurrent angioedema the day after rechallenge	To report omalizumab-associated angioedema after injections in a patient with CSU
Ertaş, 2016	[11]	Turkey	Case series within a clinical cohort	Patients with severe antihistamine-resistant CSU	46; 4 with reported reactions	CSU	300 mg monthly	Reactions occurred from 30 min to more than 12 h after the 1st to 6th doses	To report patients who developed anaphylaxis, angioedema, or urticaria flare-up during omalizumab administration
Gönül, 2016	[21]	Turkey	Case report/letter to the editor	37-year-old woman with CSU and angioedema refractory to high-dose H1-antihistamines and intermittent systemic corticosteroids	1	CSU with angioedema	300 mg every 4 weeks	Triphasic anaphylaxis occurred after the first injection, with reactions occurring within minutes and recurring at 24 and 36 h; 10-day follow-up reported	To report omalizumab-induced triphasic anaphylaxis in a patient with CSU
Savic, 2015	[28]	United Kingdom	Retrospective case note review	Patients with CSU treated with omalizumab or ciclosporin in secondary care	46 in the omalizumab cohort	CSU, with or without CIndU	Not reported in the extracted data	Not reported in the extracted data	To compare outcomes and adverse effects in patients with CSU treated with omalizumab or ciclosporin in United Kingdom secondary care

Note. CIU, chronic idiopathic urticaria; CIndU, chronic inducible urticaria; CSU, chronic spontaneous urticaria; CU, chronic urticaria; H1, histamine-1 receptor; UAS7, Urticaria Activity Score over 7 days; UCARE, Urticaria Centers of Reference and Excellence.

**Table 2:** Clinical presentation, timing, and severity of omalizumab-associated urticaria-related reactions.

Author, Year	Reference	Affected Patients	Timing in Treatment Course	Timing After Injection	Reaction Type	Clinical Presentation	Severity	Duration of Reaction	Recurrence After Later Injections
Cekic, 2026	[30]	1/61 (1.6%)	After first dose	Within 24 h	Urticaria exacerbation	Urticaria exacerbation; patient-level clinical details were not available	Not reported	Not reported	No; not repeated with subsequent doses
Lestani, 2025	[23]	1	After first administration	A few days	Serum sickness-like reaction	Arthralgia, malaise, fatigue, fever, and mildly pruritic rash	Moderate to severe; required systemic corticosteroids	More than 2 weeks; resolved after prednisone	Not applicable
Soegiharto, 2025	[14]	31/1859 (1.7%)	Not reported	Not reported	Angioedema or urticaria flare-up	Angioedema or urticaria flare-up; patient-level details were not available	Not reported	Not reported	Not reported
Chen, 2024	[13]	3/82 (3.7%)	Not reported	Not reported	Transient urticaria exacerbation	Urticaria exacerbation; patient-level details were not available	Mild; did not require treatment discontinuation	Transient	Not reported
Konstantinou, 2024	[22]	1	After dose escalation to 450 mg	1 week	CSU flare with angioedema	Deterioration of CSU with alternate-day angioedema affecting the lips	Severe; UAS7 = 35	Not reported	Not applicable
Galletta, 2023	[31]	4/9 CSU patients	Not reported	Not reported	Urticaria	Urticaria; patient-level details were not available	Mild to moderate	Not reported	Not reported
Hide, 2023	[12]	5/280 (1.8%)	Not reported	Not reported	Urticaria	Urticaria; patient-level details were not available	One urticaria event was reported as serious; overlap with the five cases was unclear	Not reported	Not reported
Öztop, 2022	[27]	1/141 (0.7%) CSU patients	After second dose	1 day	Urticaria exacerbation	Exacerbation of urticarial plaques	Severe; required corticosteroids and switch to cyclosporine	Not reported	Not applicable
Dies, 2020	[19]	1	After first and second doses	Approximately 12 h after first dose and 24 h after second dose	Possible IgE-mediated delayed-onset anaphylaxis	First dose: pyrexia and headache; second dose: severe diffuse painful urticarial rash, vomiting, mucous diarrhea, and diffuse muscular pain	Severe; poorly responsive to oral antihistamines and corticosteroids and required intravenous betamethasone	Persistent for weeks	Yes; reaction recurred after the second dose
Lapeere, 2020	[26]	1/235 (0.4%)	Not reported	Not reported	Urticaria worsening	Urticaria worsening; patient-level details were not available	Severe; required hospitalization	Not reported	Not reported
Weiss, 2020	[25]	1	After dose escalation to 300 mg	Several days	Serum sickness-like reaction	Cramping abdominal pain, fatigue, fever, inguinal lymphadenopathy, and diffuse joint aches	Moderate to severe; led to discontinuation	Improved over several months	Not applicable
Tabanlıoğlu-Onan, 2019	[29]	1/24 (4.2%)	Not reported	Not reported	Urticarial plaque at the injection area	Urticarial plaque at the injection area; patient-level details were not available	Not reported	Not reported	Not reported
Eapen, 2018	[20]	1	After the second injection; symptoms worsened after each subsequent monthly injection over the next 6 months	1 week after the second injection	Serum sickness-like reaction	Morbilliform rash, malaise, joint pain, headache, cervical and axillary lymphadenopathy, and mild thrombocytopenia; no local injection-site reaction	Moderate to severe; led to omalizumab discontinuation	Systemic symptoms resolved after discontinuation; resolved by 3-month post-discontinuation evaluation	Yes; malaise, joint pain, lymphadenopathy, and thrombocytopenia worsened with each subsequent injection
Magen, 2018	[24]	1	After the first and subsequent injections; recurred after rechallenge following discontinuation	2 days after the first injection; after each subsequent injection; 1 day after rechallenge	Omalizumab-associated angioedema, considered nonhistaminergic	Severe angioedema of the lips, face, and eyelids; baseline CSU wheals and pruritus improved markedly with omalizumab	Moderate to severe; recurrent severe facial angioedema	Each episode resolved within 2–3 days; no relapse after two subsequent injections given with tranexamic acid	Yes; recurred after subsequent injections and after rechallenge; prevented during later injections with tranexamic acid
Ertaş, 2016	[11]	4/46 (8.7%)	After first, second, fifth, or sixth dose	Less than 30 min to more than 12 h	Urticaria flare-up, angioedema, delayed anaphylaxis, or nonspecific adverse reaction	Findings ranged from angioedema involving the lips, neck, or tongue to hypotension and severe urticarial lesions	Mild to severe; some cases required epinephrine, systemic corticosteroids, and intravenous fluids	10 min to several days	Yes; recurrence occurred in two patients
Gönül, 2016	[21]	1	After the first injection	Within a few minutes; recurrent episodes at 24 and 36 h after injection	Omalizumab-induced triphasic anaphylaxis	Severe angioedema of the face, tongue, and larynx; dyspnea, tachypnea, hypotension, tachycardia, conjunctival injection, flushing, and cyanosis	Severe; required intramuscular epinephrine, methylprednisolone, and antihistamine	Initial symptoms resolved within 30 min; recurrent episodes at 24 and 36 h resolved within 20 min after treatment	Not applicable; no later omalizumab injections reported, but triphasic recurrence occurred within the same post-injection episode
Savic, 2015	[28]	2/46 (4.3%)	Not reported	2.5 h in one episode; on the day of dosing in another episode	CSU-like exacerbation/anaphylaxis-like reaction	Shortness of breath, tongue angioedema, and urticaria	Moderate to severe; required oxygen, antihistamine, hydrocortisone, epinephrine, or hospitalization	15 min to overnight	Yes; one patient had two episodes

Note. CSU, chronic spontaneous urticaria; h, hour(s); IgE, immunoglobulin E; min, minute(s); UAS7, Urticaria Activity Score over 7 days.

**Table 3:** Proposed mechanisms, differential diagnosis, and risk factors.

Author, Year	Reference	Suspected/Proposed Mechanism	Evidence Supporting Association	Alternative Explanations Considered	Risk Factors/Predisposing Factors	Method Used to Distinguish Causes	Causality/Certainty of Interpretation
Cekic, 2026	[30]	Not discussed	Temporal association only; one patient developed urticaria exacerbation within 24 h after the first dose	Not discussed	Not discussed	Not reported	Uncertain; patient-level causality assessment was not reported
Lestani, 2025	[23]	Serum sickness-like reaction	Temporal relationship after omalizumab, low C3 level, anti-dsDNA positivity, and resolution after systemic glucocorticoids	Overt autoimmune disease	Pre-existing intermittent arthralgia and possible subclinical dysimmune background	Rheumatologic consultation, laboratory testing, clinical assessment, and treatment response	Highly probable serum sickness-like reaction
Soegiharto, 2025	[14]	Nocebo effect or overlap between side effects and uncontrolled disease activity	Reported side effects were statistically associated with insufficient omalizumab response	Uncontrolled chronic urticaria symptoms overlapping with reported side effects	Female sex, angioedema, chronic inducible urticaria, worse baseline disease control, and fast response failure	Study-level statistical analysis using univariate and multivariate logistic regression	Causality could not be determined at the patient level
Chen, 2024	[13]	Not discussed	Not reported	Not discussed	Not discussed	Not reported	Uncertain; patient-level causality assessment was not reported
Konstantinou, 2024	[22]	Infection-associated flare related to parasitic infection	Worsening after dose escalation, positive parasitological findings, and complete resolution after albendazole	Spontaneous CSU flare, autoimmune/connective tissue disease, allergy, organophosphate exposure, and excipient-related hypersensitivity	Agricultural occupation, dog exposure, residence in an endemic area, and dose escalation to 450 mg	Parasitological testing, laboratory investigations, exclusion of other triggers, and response to antiparasitic therapy	Parasitic contribution strongly supported; direct omalizumab hypersensitivity was less likely
Galletta, 2023	[31]	Not specifically discussed	Urticaria was reported during omalizumab treatment	Underlying CSU	Not discussed	Not reported	Possibly related to underlying CSU; patient-level causality assessment was not available
Hide, 2023	[12]	Not specifically discussed	Urticaria was reported as an adverse event; one event was serious	Primary disease was considered for two cases initially reported as anaphylaxis with urticaria and dyspnea	Not discussed	Investigator clinical judgment	Causality for urticaria events was unclear; two anaphylaxis-like cases were judged unrelated to omalizumab
Öztop, 2022	[27]	Not discussed	Temporal relationship; urticaria plaque exacerbation occurred 1 day after the second dose	Not discussed	Not discussed	Clinical observation; no detailed workup reported	Uncertain; follow-up was incomplete
Dies, 2020	[19]	Possible IgE-mediated delayed-onset anaphylaxis; excipient hypersensitivity and serum sickness-like reaction were also considered	Temporal relationship, recurrence after rechallenge, and positive intradermal test to omalizumab	Exacerbation of primary CSU and external factors	Not reported	Skin testing, including positive intradermal test; patch and skin prick tests were negative; clinical and laboratory assessment	Possible omalizumab-associated delayed-onset anaphylaxis
Lapeere, 2020	[26]	Not discussed	Urticaria worsening was reported as a severe adverse event requiring hospitalization	Not discussed	Not discussed	Not reported	Unknown association with omalizumab
Weiss, 2020	[25]	Serum sickness-like reaction	Temporal relationship after dose escalation and gradual improvement after omalizumab discontinuation	Infection, autoimmune disease, and malignancy	Dose escalation from 150 mg to 300 mg	Laboratory tests, lymph node biopsy, and subspecialty evaluation	Suspected serum sickness-like reaction
Tabanlıoğlu-Onan, 2019	[29]	Not discussed	Urticarial plaque was reported at the injection area	Not discussed	Not discussed	Not reported	Uncertain; patient-level causality assessment was not reported
Eapen, 2018	[20]	Serum sickness-like reaction secondary to omalizumab	Reaction began after omalizumab initiation, developed 1 week after the second injection, worsened with each subsequent injection, and resolved after omalizumab discontinuation; clinical features included morbilliform rash, malaise, arthralgia, lymphadenopathy, and mild thrombocytopenia	Infection and estrogen/progesterone sensitivity were considered; hormonal sensitivity may have contributed to baseline urticaria/angioedema, but was considered less likely to explain the serum sickness-like reaction because the reaction began only after omalizumab initiation and resolved after discontinuation	Pediatric patient with CIU/CSU and angioedema; possible hormonal contribution to baseline urticaria/angioedema; repeated exposure despite systemic symptoms	Clinical history, temporal relationship, dechallenge after omalizumab discontinuation, consideration of alternative causes, and symptom resolution after stopping omalizumab	Most consistent with serum sickness-like reaction to omalizumab; cause–effect relationship could not be completely confirmed
Magen, 2018	[24]	Omalizumab-related nonhistaminergic angioedema; possible bradykinin-mediated pathway related to mast-cell heparin release and Factor XII activation	New-onset severe angioedema developed after omalizumab despite absence of baseline angioedema; episodes recurred after subsequent injections and after rechallenge; angioedema did not occur during urticaria relapse after omalizumab discontinuation; later episodes were prevented with tranexamic acid	Baseline CSU-associated angioedema, histaminergic angioedema, and complement-mediated angioedema were considered	Severe antihistamine-resistant CSU; recurrent exposure and rechallenge; possible susceptibility to nonhistaminergic angioedema	C1-INH, complement factor 4, and tryptase testing at baseline and during episodes; dechallenge/rechallenge observation; clinical response to tranexamic acid	Probable omalizumab-associated nonhistaminergic angioedema; proposed mechanism remained hypothetical
Ertaş, 2016	[11]	Delayed hypersensitivity, possible excipient-related reaction, acute adverse reaction, drug ineffectiveness, or spontaneous CSU flare	Temporal association with omalizumab and recurrence after rechallenge in some patients	Spontaneous CSU flare, drug ineffectiveness, and exacerbation after discontinuation of other medications	Severe antihistamine-resistant CSU	Timing-based clinical assessment, including consideration of reactions within 2 h versus later reactions	Mixed interpretation; suspected adverse reaction in some cases, with disease flare or ineffectiveness considered in others
Gönül, 2016	[21]	Omalizumab-induced triphasic anaphylaxis	Severe multisystem reaction occurred within minutes after the first injection, with face, tongue, and laryngeal angioedema, dyspnea, hypotension, tachycardia, flushing, conjunctival injection, and cyanosis; additional anaphylactic episodes recurred at 24 and 36 h after injection	Alternative explanations were not discussed in detail; underlying CSU with angioedema was present, but the immediate timing and systemic features supported anaphylaxis	CSU with angioedema; first omalizumab exposure; no previous history of anaphylaxis was reported	Clinical diagnosis based on immediate temporal relationship, multisystem involvement, response to epinephrine/corticosteroid/antihistamine treatment, and short-term follow-up	Probable omalizumab-induced triphasic anaphylaxis; formal causality assessment was not reported
Savic, 2015	[28]	Severe CSU exacerbation mimicking anaphylaxis rather than true omalizumab-induced anaphylaxis	Clinical history of similar reactions before omalizumab treatment	Omalizumab-induced anaphylaxis	Complex and severe CSU, including previous anaphylaxis-like episodes before omalizumab	Retrospective case-note review and investigator clinical interpretation	Most likely unrelated to omalizumab and attributed to underlying CSU activity

Note. anti-dsDNA, anti-double-stranded DNA; C1-INH, C1 esterase inhibitor; C3, complement component 3; CIU, chronic idiopathic urticaria; CSU, chronic spontaneous urticaria; h, hour(s); IgE, immunoglobulin E.

**Table 4:** Management, treatment decisions, outcomes, and evidence gaps.

**Author, Year**	**Reference**	Acute Management	Omalizumab Treatment Decision	Rechallenge/Dose Modification	Alternative or Subsequent Treatment	Clinical Outcome/Prognosis	Discontinuation Due to Reaction	Cross-Reaction/Tolerance to Other Biologics	Key Evidence Gap
Cekic, 2026	[30]	Not reported	Continued	Subsequent doses were administered	Not reported	Transient; complication did not recur with subsequent doses	No	Not reported	Patient-level acute management and detailed clinical course were not reported
Lestani, 2025	[23]	Paracetamol, ibuprofen, antihistamines, followed by systemic prednisone	Discontinued	Not rechallenged	Daily antihistamines	Complete resolution of serum sickness-like reaction; CSU remained active	Yes	Not reported	Cross-reaction or tolerance to other biologics was not reported
Soegiharto, 2025	[14]	Patient-level details not available	Unclear	Patient-level details not available	Patient-level details not available	Patient-level details not available	Unclear	Not reported	Patient-level management, discontinuation attribution, rechallenge, and outcomes were not reported
Chen, 2024	[13]	Patient-level details not available	Continued	Continued	Not reported	Transient urticaria exacerbation resolved	No	Not reported	Patient-level acute management, duration, and recurrence were not reported
Konstantinou, 2024	[22]	Omalizumab discontinuation, levocetirizine, methylprednisolone, and albendazole	Discontinued	Not rechallenged	Albendazole and levocetirizine	Complete CSU resolution after antiparasitic treatment	Yes	Not reported	Cross-reaction or tolerance to other biologics was not reported
Galletta, 2023	[31]	Patient-level details not available	Continued	Continued	Not reported	Patient-level outcomes not available	No	Not reported	Patient-level acute management and specific outcomes for urticaria events were not reported
Hide, 2023	[12]	Patient-level details not available	Unclear for standard urticaria events	Patient-level details not available	Patient-level details not available	Patient-level outcomes not available for urticaria events	Unclear	Not reported	Management and discontinuation were not linked specifically to the five urticaria adverse events
Öztop, 2022	[27]	Systemic corticosteroids	Switched from omalizumab	Not rechallenged	Cyclosporine	Unknown; patient was lost to follow-up	Yes	Not reported	Final outcome after switching treatment was unknown
Dies, 2020	[19]	Intravenous betamethasone, chlorpheniramine, ranitidine, and saline	Discontinued after recurrent reaction	Rechallenged after first reaction; recurrent/worse reaction occurred after second dose	Cyclosporine	Persistent urticarial lesions at 50 days	Yes	Not reported	Long-term outcome and biologic cross-reactivity were not reported
Lapeere, 2020	[26]	Hospitalization reported	Patient-level details not available	Patient-level details not available	Patient-level details not available	Patient-level outcomes not available	Unclear	Not reported	Specific acute medications, treatment decision, and outcome were not reported
Weiss, 2020	[25]	Omalizumab was held; acute medications not reported	Discontinued	Not rechallenged	Not reported	Symptoms gradually improved over several months	Yes	Not reported	Acute pharmacologic management and alternative treatment were not reported
Tabanlıoğlu-Onan, 2019	[29]	Patient-level details not available	Continued	Continued	Not reported	Patient-level outcomes for the injection-site urticarial plaque were not available	No	Not reported	Patient-level management and duration of reaction were not reported
Eapen, 2018	[20]	No acute medication specifically for the serum sickness-like reaction was reported; patient had been receiving H1 and H2 blockers, montelukast, doxepin, and later sulfasalazine	Discontinued	Not rechallenged after discontinuation	Cyclosporine was recommended but not started; norethindrone was later started for suspected hormonal contribution to recurrent urticaria/angioedema	Malaise, joint pain, and lymphadenopathy resolved after omalizumab discontinuation; pruritus and angioedema recurred, then resolved after norethindrone; patient remained free of hives, pruritus, and angioedema at 5 months after starting norethindrone	Yes	Not reported	Formal causality assessment, acute management details, and biologic cross-reactivity/tolerance were not reported
Magen, 2018	[24]	Tranexamic acid 1 g three times daily for 7 days after each omalizumab injection; continued fexofenadine	Initially continued; later discontinued after 6 months; subsequently restarted	Rechallenge occurred after discontinuation and caused recurrent severe angioedema; later omalizumab injections were continued with tranexamic acid prophylaxis	Tranexamic acid prophylaxis; fexofenadine continued	Angioedema episodes resolved within 2–3 days; no relapse occurred after two subsequent omalizumab injections given with tranexamic acid; CSU wheals and pruritus improved markedly with omalizumab	Temporarily yes; later restarted	Not reported	Long-term outcome, recurrence beyond two prophylaxis-supported injections, and biologic cross-reactivity/tolerance were not reported
Ertaş, 2016	[11]	Oral antihistamines, systemic corticosteroids, intravenous fluids, and subcutaneous epinephrine	Discontinued in affected patients	Rechallenge after initially milder reactions led to more severe subsequent reactions in some patients	Cyclosporine in one case; antihistamines and corticosteroids in others	Resolution within 10 min to 3 days	Yes	Not reported	Cross-reaction or tolerance to other biologics was not reported
Gönül, 2016	[21]	Intramuscular adrenaline, methylprednisolone, and pheniramine maleate; repeat adrenaline was required during the initial episode; the same regimen was used for recurrent episodes	Discontinued/No further injections reported	Not rechallenged; triphasic recurrence occurred at 24 and 36 h after the same first injection	Not reported	Initial symptoms resolved within 30 min; recurrent episodes resolved within 20 min after treatment; no further anaphylaxis was reported at 10-day follow-up	Yes	Not reported	Long-term outcome, formal causality assessment, and tolerance to other biologics were not reported
Savic, 2015	[28]	Oxygen, antihistamines, hydrocortisone, self-administered adrenaline, and hospital admission in selected episodes	Continued	Continued despite reactions	Not reported	Acute resolution within 15 min to overnight	No	Not reported	Cross-reaction or tolerance to other biologics was not reported

Note. CSU, chronic spontaneous urticaria; g, gram(s); H1, histamine-1 receptor; H2, histamine-2 receptor; h, hour(s); min, minute(s).

## Data Availability

All data supporting the findings of this review are included in the manuscript. No additional datasets were generated or analyzed.

## Supplementary Materials

The following supporting information can be downloaded at: https://www.mdpi.com/article/10.3390/life16071124/s1, Checklist S1: PRISMA-ScR checklist; Supplementary Table S1: Draft data-charting form used for data extraction; Supplementary Table S2: Original and supplementary sensitivity search strategies; Supplementary Table S3: Structured WHO-UMC-informed causality-plausibility assessment for case-based reports.

## Abbreviations

The following abbreviations are used in this manuscript:

ACE	Angiotensin-converting enzyme
ADR	Adverse drug reaction
CIU	Chronic idiopathic urticaria
CIndU	Chronic inducible urticaria
CSU	Chronic spontaneous urticaria
CU	Chronic urticaria
DLQI	Dermatology Life Quality Index
DOAJ	Directory of Open Access Journals
EMA	European Medicines Agency
FAERS	Food and Drug Administration Adverse Event Reporting System
H1	Histamine-1 receptor
H2	Histamine-2 receptor
IgE	Immunoglobulin E
INPLASY	International Platform of Registered Systematic Review and Meta-analysis Protocols
IV	Intravenous
JBI	Joanna Briggs Institute
PCC	Population–concept–context
PRISMA	Preferred Reporting Items for Systematic Reviews and Meta-Analyses
PRISMA-ScR	Preferred Reporting Items for Systematic Reviews and Meta-Analyses extension for Scoping Reviews
UAS7	Urticaria Activity Score over 7 days
UCARE	Urticaria Centers of Reference and Excellence
WHO ICTRP	World Health Organization International Clinical Trials Registry Platform
WHO-UMC	World Health Organization–Uppsala Monitoring Centre

## References

[1] Kolkhir P., Bonnekoh H., Metz M., Maurer M. (2024). Chronic spontaneous urticaria: A review. JAMA.

[2] Zhao Z., Huang Y., Chen D., Wang Y. (2025). Global health implications of urticaria burden (1990–2021): A comprehensive analysis of trends across nations and regions. Arch. Med. Sci..

[3] Gonçalo M., Gimenéz-Arnau A., Al-Ahmad M., Ben-Shoshan M., Bernstein J.A., Ensina L.F., Fomina D., Galvàn C.A., Godse K., Grattan C. (2021). The global burden of chronic urticaria for the patient and society. Br. J. Dermatol..

[4] Zuberbier T., Abdul Hameed Ansari Z., Abdul Latiff A.H., Abuzakouk M.M., Agcaoili-De Jesus M.S., Agondi R.C., Al-Ahmad M., Alangari A.A., Alhameli H., Alonso Bello C.D. (2026). The international guideline for the definition, classification, diagnosis and management of urticaria. Allergy.

[5] Metz M., Vadasz Z., Kocatürk E., Giménez-Arnau A.M. (2020). Omalizumab Updosing in Chronic Spontaneous Urticaria: An Overview of Real-World Evidence. Clin. Rev. Allergy Immunol..

[6] Sabroe R. (2014). Commentary: Omalizumab for the treatment of chronic idiopathic or spontaneous urticaria. Br. J. Dermatol..

[7] Easthope S., Jarvis B. (2001). Omalizumab. Drugs.

[8] Casale T.B., Gimenez-Arnau A.M., Bernstein J.A., Holden M., Zuberbier T., Maurer M. (2023). Omalizumab for patients with chronic spontaneous urticaria: A narrative review of current status. Dermatol. Ther..

[9] Jia H.-X., He Y.-L. (2020). Efficacy and Safety of Omalizumab for Chronic Spontaneous Urticaria: A Systematic Review and Meta-Analysis of Randomized Controlled Trials. Am. J. Ther..

[10] Pongdee T., Li J.T. (2025). Omalizumab safety concerns. J. Allergy Clin. Immunol..

[11] Ertaş R., Özyurt K., Yildiz S., Ulaş Y., Turasan A., Avci A. (2016). Adverse reaction to omalizumab in patients with chronic urticaria: Flare up or ineffectiveness?. Iran. J. Allergy Asthma Immunol..

[12] Hide M., Fukunaga A., Suzuki T., Nakamura N., Kimura M., Sasajima T., Kiriyama J., Igarashi A. (2023). Real-world safety and effectiveness of omalizumab in Japanese patients with chronic spontaneous urticaria: A post-marketing surveillance study. Allergol. Int..

[13] Chen J., Ou S., Wu W., Zou H., Li H., Zhu H. (2024). Omalizumab in Chronic Spontaneous Urticaria: A Real-World Study on Effectiveness, Safety and Predictors of Treatment Outcome. Clin. Cosmet. Investig. Dermatol..

[14] Soegiharto R., Van der Wind E., Alizadeh Aghdam M., Sørensen J.A., Van Lindonk E., Bulut Demir F., Mohammad Porras N., Matsuo Y., Kiefer L., Knulst A.C. (2025). Spectrum and Impact of Reported Side Effects of Omalizumab in Patients With Chronic Urticaria: A Long-Term Multicentre Real-World Study. Clin. Exp. Allergy.

[15] Pollock D., Tricco A.C., Peters M.D., Mclnerney P.A., Khalil H., Godfrey C.M., Alexander L.A., Munn Z. (2022). Methodological quality, guidance, and tools in scoping reviews: A scoping review protocol. JBI Evid. Synth..

[16] Tricco A.C., Lillie E., Zarin W., O’Brien K.K., Colquhoun H., Levac D., Moher D., Peters M.D., Horsley T., Weeks L. (2018). PRISMA extension for scoping reviews (PRISMA-ScR): Checklist and explanation. Ann. Intern. Med..

[17] Page M.J., McKenzie J.E., Bossuyt P.M., Boutron I., Hoffmann T.C., Mulrow C.D., Shamseer L., Tetzlaff J.M., Akl E.A., Brennan S.E. (2021). The PRISMA 2020 statement: An updated guideline for reporting systematic reviews. BMJ.

[18] Shukla A.K., Jhaj R., Misra S., Ahmed S.N., Nanda M., Chaudhary D. (2021). Agreement between WHO-UMC causality scale and the Naranjo algorithm for causality assessment of adverse drug reactions. J. Fam. Med. Prim. Care.

[19] Dies L., Sernicola A., Magri F., Chello C., Paolino G., Carnicelli G., Faina V., Nencini F., Grieco T. (2020). A severe adverse reaction to omalizumab therapy in chronic spontaneous urticaria. Dermatol. Ther..

[20] Eapen A., Kloepfer K.M. (2018). Serum sickness-like reaction in a pediatric patient using omalizumab for chronic spontaneous urticaria. Pediatr. Allergy Immunol..

[21] Gönül M., Özenergün Bittacı A., Ergin C. (2016). Omalizumab-induced triphasic anaphylaxis in a patient with chronic spontaneous urticaria. J. Eur. Acad. Dermatol. Venereol..

[22] Konstantinou G.N., Podder I. (2024). Paradoxical Worsening of Chronic Spontaneous Urticaria Following Omalizumab Administration: The Missing Link. Cureus.

[23] Lestani V., Zelin E., Stinco G., Errichetti E. (2025). Serum sickness-like reaction after omalizumab administration: A case report and short literature review. J. Dtsch. Dermatol. Ges..

[24] Magen E., Chikovani T. (2018). Development of angio-oedema after omalizumab injections in a patient with chronic spontaneous urticaria. Clin. Exp. Dermatol..

[25] Weiss S.L., Smith D.M. (2020). A Case of Serum Sickness-Like Reaction in an Adult Treated with Omalizumab. Mil. Med..

[26] Lapeere H., Baeck M., Stockman A., Sabato V., Grosber M., Moutschen M., Lambert J., Vandebuerie L., de Montjoye L., Rabijns H. (2020). A retrospective analysis omalizumab treatment patterns in patients with chronic spontaneous urticaria: A real-world study in Belgium. J. Eur. Acad. Dermatol. Venereol..

[27] Öztop N., Demir S., Beyaz Ş., Tüzer Ö.C., Çolakoğlu B., Büyüköztürk S., Gelincik A. (2022). Omalizumab in Practice: Ten-Year Experience of A Tertiary Referral Allergy Centre. Asthma Allergy Immunol..

[28] Savic S., Marsland A., McKay D., Ardern-Jones M.R., Leslie T., Somenzi O., Baldock L., Grattan C. (2015). Retrospective case note review of chronic spontaneous urticaria outcomes and adverse effects in patients treated with omalizumab or ciclosporin in UK secondary care. Allergy Asthma Clin. Immunol..

[29] Tabanlioğlu-Onan D., Öktem A., Yalçin B., Artüz F. (2019). Efficacy of omalizumab in treatment-resistant chronic spontaneous urticaria. Asthma Allergy Immunol..

[30] Cekic S., Canitez Y., Ozceker D., Uysal P., Ozdemir O., Filiz S., Bologur H., Karali Y., Yuksel H., Sapan N. (2026). The Efficacy of Different Dosing Regimens of Omalizumab in Children and Adolescents with Chronic Spontaneous Urticaria Based on Real-Life Data. Int. Arch. Allergy Immunol..

[31] Galletta F., Caminiti L., Lugarà C., Foti Randazzese S., Barraco P., D’Amico F., Irrera P., Crisafulli G., Manti S. (2023). Long-Term Safety of Omalizumab in Children with Asthma and/or Chronic Spontaneous Urticaria: A 4-Year Prospective Study in Real Life. J. Pers. Med..

[32] Jackson K., Bahna S.L. (2020). Hypersensitivity and adverse reactions to biologics for asthma and allergic diseases. Expert Rev. Clin. Immunol..

[33] Kocaturk E., Saini S.S., Rubeiz C.J., Bernstein J.A. (2022). Existing and investigational medications for refractory chronic spontaneous urticaria: Safety, adverse effects, and monitoring. J. Allergy Clin. Immunol. Pract..

[34] Maurer M., Casale T.B., Saini S.S., Ben-Shoshan M., Giménez-Arnau A.M., Bernstein J.A., Yagami A., Stjepanovic A., Radin A., Staudinger H.W. (2024). Dupilumab in patients with chronic spontaneous urticaria (LIBERTY-CSU CUPID): Two randomized, double-blind, placebo-controlled, phase 3 trials. J. Allergy Clin. Immunol..

[35] Mastorino L., Ortoncelli M., Virginia B., Rolla G., Avallone G., Cavaliere G., Riccardo V., Quaglino P., Ribero S. (2021). Dupilumab-induced Urticaria. Dermatol. Ther..

[36] Xu J., Shih J., Kalangara M. (2021). Delayed onset localized urticarial reactions to dupilumab. J. Allergy Clin. Immunol..

[37] Fritz A.L., Lacy F.A., Morrell D.S. (2021). Angioedema: A potential complication of dupilumab in atopic dermatitis. Pediatr. Dermatol..

[38] Burhan M., Ashraf S., Ali A., Shahid I., Ahmed J., Shah M.S.U., Bhagwan Das N., Nashwan A.J. (2025). Safety and Efficacy of Remibrutinib for Chronic Spontaneous Urticaria: A Systematic Review and Meta-Analysis. Int. Arch. Allergy Immunol..

[39] Chen C.B., Hsu T.H., Chen I.W., Chang P.N., Wang C.W., Chung W.H. (2025). Immune checkpoint inhibitor-related urticaria characterized by a distinct autoimmune and autoallergic signatures. Dermatol. Sin..

[40] Yilmaz Topal O., Kose V., Acar B., Bayrakci U.S., Ozyoruk D., Hizal G., Karaatmaca B., Toyran M., Yarali H.N., Ozbek N.Y. (2021). Evaluation of hypersensitivity reactions in pediatric patients using biological drugs. Int. Arch. Allergy Immunol..

[41] Sala-Cunill A., Luengo O., Cardona V. (2019). Biologics and anaphylaxis. Curr. Opin. Allergy Clin. Immunol..

[42] Vultaggio A., Maggi E., Matucci A. (2011). Immediate adverse reactions to biologicals: From pathogenic mechanisms to prophylactic management. Curr. Opin. Allergy Clin. Immunol..

[43] Grumach A.S., Staubach-Renz P., Villa R.C., Diez-Zuluaga S., Reese I., Lumry W.R. (2021). Triggers of exacerbation in chronic urticaria and recurrent angioedema—Prevalence and relevance. J. Allergy Clin. Immunol. Pract..

[44] Kolkhir P., Muñoz M., Asero R., Ferrer M., Kocatürk E., Metz M., Xiang Y.-K., Maurer M. (2022). Autoimmune chronic spontaneous urticaria. J. Allergy Clin. Immunol..

[45] Rönsch H., Berndt K., Bauer A. (2021). Treatment satisfaction in chronic urticaria during guideline-based therapy. JDDG J. Ger. Soc. Dermatol..

[46] Sánchez J., Amaya E., Acevedo A., Celis A., Caraballo D., Cardona R. (2017). Prevalence of Inducible Urticaria in Patients with Chronic Spontaneous Urticaria: Associated Risk Factors. J. Allergy Clin. Immunol. Pract..

[47] Genentech I. (2024). XOLAIR (Omalizumab) Injection, for Subcutaneous Use.

[48] Steele R.H., Limaye S., Cleland B., Chow J., Suranyi M.G. (2005). Hypersensitivity reactions to the polysorbate contained in recombinant erythropoietin and darbepoietin. Nephrology.

[49] Bartels C.L., Sanz C., Stec R., Coulter D.W. (2012). Parenteral nutrition–induced hypersensitivity in an adolescent. J. Parenter. Enter. Nutr..

[50] Singh S.K., Mahler H.C., Hartman C., Stark C.A. (2018). Are Injection Site Reactions in Monoclonal Antibody Therapies Caused by Polysorbate Excipient Degradants?. J. Pharm. Sci..

[51] Caballero M.L., Quirce S. (2020). Delayed hypersensitivity reactions caused by drug excipients: A literature review. J. Investig. Allergol. Clin. Immunol..

[52] Coors E.A., Seybold H., Merk H.F., Mahler V. (2005). Polysorbate 80 in medical products and nonimmunologic anaphylactoid reactions. Ann. Allergy Asthma Immunol..

[53] Stone C.A., Liu Y., Relling M.V., Krantz M.S., Pratt A.L., Abreo A., Hemler J.A., Phillips E.J. (2019). Immediate Hypersensitivity to Polyethylene Glycols and Polysorbates: More Common Than We Have Recognized. J. Allergy Clin. Immunol. Pract..

[54] Wenande E., Garvey L.H. (2016). Immediate-type hypersensitivity to polyethylene glycols: A review. Clin. Exp. Allergy.

[55] Wylon K., Dölle S., Worm M. (2016). Polyethylene glycol as a cause of anaphylaxis. Allergy Asthma Clin. Immunol..

[56] Co-Minh H., Demoly P., Guillot B., Raison-Peyron N. (2007). Anaphylactic shock after oral intake and contact urticaria due to polyethylene glycols. Allergy.

[57] Imbalzano E., Casciaro M., Quartuccio S., Minciullo P.L., Cascio A., Calapai G., Gangemi S. (2016). Association between urticaria and virus infections: A systematic review. Allergy Asthma Proc..

[58] Liu Z., Al-Quran L., Tong J., Cao X. (2023). Analysis of clinical features and inflammatory-related molecules with the disease in acute infectious urticaria. Arch. Dermatol. Res..

[59] Sackesen C., Sekerel B.E., Orhan F., Kocabas C.N., Tuncer A., Adalioglu G. (2004). The etiology of different forms of urticaria in childhood. Pediatr. Dermatol..

[60] Wedi B., Raap U., Kapp A. (2004). Chronic urticaria and infections. Curr. Opin. Allergy Clin. Immunol..

[61] Arik Yilmaz E., Karaatmaca B., Sackesen C., Sahiner U.M., Cavkaytar O., Sekerel B.E., Soyer O. (2016). Parasitic infections in children with chronic spontaneous urticaria. Int. Arch. Allergy Immunol..

[62] Kolkhir P., Balakirski G., Merk H.F., Olisova O., Maurer M. (2016). Chronic spontaneous urticaria and internal parasites—A systematic review. Allergy.

[63] Matucci A., Parronchi P., Rossi O., Vultaggio A., Deleonardi G., Orsi A., Maggi E., Romagnani S. (2004). A case of chronic urticaria due to Dirofilaria infestation. Allergol. Int..

[64] Minciullo P.L., Cascio A., Gangemi S. (2018). Association between urticaria and nematode infections. Allergy Asthma Proc..

[65] Pasqui A., Savini E., Saletti M., Guzzo C., Puccetti L., Auteri A. (2004). Chronic urticaria and Blastocystis hominis infection. A case report. Eur. Rev. Med. Pharmacol. Sci..

[66] Bracken S.J., Abraham S., MacLeod A.S. (2019). Autoimmune theories of chronic spontaneous urticaria. Front. Immunol..

[67] Confino-Cohen R., Chodick G., Shalev V., Leshno M., Kimhi O., Goldberg A. (2012). Chronic urticaria and autoimmunity: Associations found in a large population study. J. Allergy Clin. Immunol..

[68] Kolkhir P., Pogorelov D., Olisova O., Maurer M. (2016). Comorbidity and pathogenic links of chronic spontaneous urticaria and systemic lupus erythematosus—A systematic review. Clin. Exp. Allergy.

[69] Krause K., Grattan C., Bindslev-Jensen C., Gattorno M., Kallinich T., De Koning H., Lachmann H., Lipsker D., Navarini A., Simon A. (2012). How not to miss autoinflammatory diseases masquerading as urticaria. Allergy.

[70] Mathelier-Fusade P. (2006). Drug-induced urticarias. Clin. Rev. Allergy Immunol..

[71] Inomata N. (2012). Recent advances in drug-induced angioedema. Allergol. Int..

[72] Kowalski M.L., Woessner K., Sanak M. (2015). Approaches to the diagnosis and management of patients with a history of nonsteroidal anti-inflammatory drug–related urticaria and angioedema. J. Allergy Clin. Immunol..

[73] Fong A.T., Jacob J.G., Carroll B., Fernando S.L. (2021). Acute dystonic reaction mimicking angioedema secondary to metoclopramide ingestion. Emerg. Med. Australas..

[74] Gulec M., Caliskaner Z., Kartal O., Erel F., Karaayvaz M. (2007). Not all ACE inhibitor related angioedema is always evident: A case which is misdiagnosed as panic attack and speech disorder. Allergol. Immunopathol..

[75] Carter M.C., Desai A., Komarow H.D., Bai Y., Clayton S.T., Clark A.S., Ruiz-Esteves K.N., Long L.M., Cantave D., Wilson T.M. (2018). A distinct biomolecular profile identifies monoclonal mast cell disorders in patients with idiopathic anaphylaxis. J. Allergy Clin. Immunol..

[76] Gülen T., Akin C., Bonadonna P., Siebenhaar F., Broesby-Olsen S., Brockow K., Niedoszytko M., Nedoszytko B., Oude Elberink H.N., Butterfield J.H. (2021). Selecting the right criteria and proper classification to diagnose mast cell activation syndromes: A critical review. J. Allergy Clin. Immunol. Pract..

[77] Kim H.L., Leigh R., Becker A. (2010). Omalizumab: Practical considerations regarding the risk of anaphylaxis. Allergy Asthma Clin. Immunol..

[78] Lieberman P.L., Jones I., Rajwanshi R., Rosén K., Umetsu D.T. (2017). Anaphylaxis associated with omalizumab administration: Risk factors and patient characteristics. J. Allergy Clin. Immunol..

[79] Mandel V.D., Guanti M.B., Liberati S., Demonte A., Pellacani G., Pepe P. (2018). Omalizumab in Chronic Spontaneous Urticaria Refractory to Conventional Therapy: An Italian Retrospective Clinical Analysis with Suggestions for Long-Term Maintenance Strategies. Dermatol. Ther..

[80] Ozbagcivan O., Akarsu S., Ilknur T., Fetil E. (2018). Urticaria and angioedema as possible reactions of omalizumab. Bras. Dermatol..

[81] Gan H., Wang L., Fu W., Zhang J., Yu M., Liu G. (2019). Rapid subcutaneous desensitization for the management of delayed hypersensitivity reactions to omalizumab: A case report. J. Clin. Pharm. Ther..

