# Supplementary material for: Omalizumab-Associated Post-Injection Urticaria Exacerbation and Urticaria-Related Adverse Reactions During Treatment for Chronic Urticaria: A Scoping Review
Source: Life (Basel). 2026 Jul 6;16(7):1124. doi: 10.3390/life16071124 (PMC13413090; doi:10.3390/life16071124)
Supplement: Supplementary file 1 [file life-16-01124-s001.zip › life-4389978-supplementary.pdf]

Checklist S1. Preferred Reporting Items for Systematic reviews and Meta-Analyses extension for Scoping Reviews (PRISMA-ScR) Checklist

SECTION	ITEM	PRISMA-ScR CHECKLIST ITEM	REPORTED ON PAGE #
		**TITLE**	
Title	1	Identify the report as a scoping review.	1
		**ABSTRACT**	
Structured summary	2	Provide a structured summary that includes (as applicable): background, objectives, eligibility criteria, sources of evidence, charting methods, results, and conclusions that relate to the review questions and objectives.	1
		**INTRODUCTION**	
Rationale	3	Describe the rationale for the review in the context of what is already known. Explain why the review questions/objectives lend themselves to a scoping review approach.	2-3
Objectives	4	Provide an explicit statement of the questions and objectives being addressed with reference to their key elements (e.g., population or participants, concepts, and context) or other relevant key elements used to conceptualize the review questions and/or objectives.	3-4
		**METHODS**	
Protocol and registration	5	Indicate whether a review protocol exists; state if and where it can be accessed (e.g., a Web address); and if available, provide registration information, including the registration number.	3-4
Eligibility criteria	6	Specify characteristics of the sources of evidence used as eligibility criteria (e.g., years considered, language, and publication status), and provide a rationale.	4
Information sources*	7	Describe all information sources in the search (e.g., databases with dates of coverage and contact with authors to identify additional sources), as well as the date the most recent search was executed.	4-5
Search	8	Present the full electronic search strategy for at least 1 database, including any limits used, such that it could be repeated.	5
Selection of sources of evidence†	9	State the process for selecting sources of evidence (i.e., screening and eligibility) included in the scoping review.	5
Data charting process‡	10	Describe the methods of charting data from the included sources of evidence (e.g., calibrated forms or forms that have been tested by the team before their use, and whether data charting was done independently or in duplicate) and any processes for obtaining and confirming data from investigators.	6
Data items	11	List and define all variables for which data were sought and any assumptions and simplifications made.	6
Critical appraisal of individual sources of evidence§	12	If done, provide a rationale for conducting a critical appraisal of included sources of evidence; describe the methods used and how this information was used in any data synthesis (if appropriate).	N/A; rationale on p. 6
Synthesis of results	13	Describe the methods of handling and summarizing the data that were charted.	6-7
		**RESULTS**	
Selection of sources of evidence	14	Give numbers of sources of evidence screened, assessed for eligibility, and included in the review, with reasons for	7

		exclusions at each stage, ideally using a flow diagram.	
Characteristics of sources of evidence	15	For each source of evidence, present characteristics for which data were charted and provide the citations.	7-12
Critical appraisal within sources of evidence	16	If done, present data on critical appraisal of included sources of evidence (see item 12).	N/A; rationale on p.6
Results of individual sources of evidence	17	For each included source of evidence, present the relevant data that were charted that relate to the review questions and objectives.	8-25
Synthesis of results	18	Summarize and/or present the charting results as they relate to the review questions and objectives.	12-25
**DISCUSSION**			
Summary of evidence	19	Summarize the main results (including an overview of concepts, themes, and types of evidence available), link to the review questions and objectives, and consider the relevance to key groups.	26-33
Limitations	20	Discuss the limitations of the scoping review process.	33
Conclusions	21	Provide a general interpretation of the results with respect to the review questions and objectives, as well as potential implications and/or next steps.	33-34
**FUNDING**			
Funding	22	Describe sources of funding for the included sources of evidence, as well as sources of funding for the scoping review. Describe the role of the funders of the scoping review.	34

JBI = Joanna Briggs Institute; PRISMA-ScR = Preferred Reporting Items for Systematic reviews and Meta-Analyses extension for Scoping Reviews.

* Where *sources of evidence* (see second footnote) are compiled from, such as bibliographic databases, social media platforms, and Web sites.

† A more inclusive/heterogeneous term used to account for the different types of evidence or data sources (e.g., quantitative and/or qualitative research, expert opinion, and policy documents) that may be eligible in a scoping review as opposed to only studies. This is not to be confused with *information sources* (see first footnote).

‡ The frameworks by Arksey and O'Malley (6) and Levac and colleagues (7) and the JBI guidance (4, 5) refer to the process of data extraction in a scoping review as data charting.

§ The process of systematically examining research evidence to assess its validity, results, and relevance before using it to inform a decision. This term is used for items 12 and 19 instead of "risk of bias" (which is more applicable to systematic reviews of interventions) to include and acknowledge the various sources of evidence that may be used in a scoping review (e.g., quantitative and/or qualitative research, expert opinion, and policy document).

Supplementary Table S1. Draft data-charting form used for data extraction

Domain	Extracted item
Study identification	First author, year of publication, country, journal, reference number
Study characteristics	Study design, source type, clinical setting, study aim or purpose
Population	Sample size, age group, sex, type of chronic urticaria, baseline disease severity where reported
Omalizumab treatment	Dose, dosing interval, treatment duration before adverse reaction, dose escalation, number of injections before reaction
Adverse-reaction characteristics	Type of urticaria-related or hypersensitivity-type adverse reaction, timing after injection, timing in treatment course, clinical presentation, severity, duration, recurrence
Mechanism and causality	Proposed mechanism, evidence supporting association with omalizumab, alternative explanations considered, risk factors or predisposing factors, method used to distinguish causes, causality interpretation
Management	Acute treatment, omalizumab continuation or discontinuation, rechallenge or dose modification, alternative or subsequent treatment
Outcomes	Clinical outcome, recurrence after subsequent injections, long-term disease control, cross-reaction or tolerance to other biologics or targeted therapies where reported
Evidence gaps	Missing patient-level information, unclear causality, unreported management, unreported rechallenge, unreported long-term outcome

Supplementary Table S2. Original and supplementary sensitivity search strategies

Database	Search strategy	Records retrieved
**Original registered search**		
Scopus	TITLE-ABS-KEY ((omalizumab OR xolair OR "anti-IgE" OR "anti IgE" OR "anti-immunoglobulin E" OR "anti immunoglobulin E") AND ("chronic spontaneous urticaria" OR "chronic idiopathic urticaria" OR "chronic urticaria" OR CSU OR CIU) AND (exacerbation OR flare OR flares OR worsening OR aggravation OR "urticaria flare" OR "urticarial flare" OR "disease flare" OR "disease exacerbation" OR "post-injection" OR "post injection" OR "after injection" OR "following injection" OR "delayed reaction" OR "delayed hypersensitivity" OR "paradoxical reaction" OR "paradoxical exacerbation" OR "injection reaction" OR "injection-site reaction" OR "adverse event" OR "adverse reaction" OR "adverse drug reaction" OR "treatment-emergent")) AND (PUBYEAR < 2027) AND (LIMIT-TO (DOCTYPE , "ar") OR LIMIT-TO (DOCTYPE , "le") OR LIMIT-TO (DOCTYPE , "no")) AND (LIMIT-TO (LANGUAGE , "English"))	346
PubMed	(omalizumab[Title/Abstract] OR xolair[Title/Abstract] OR "anti-IgE"[Title/Abstract] OR "anti IgE"[Title/Abstract] OR "anti-immunoglobulin E"[Title/Abstract] OR "anti immunoglobulin E"[Title/Abstract]) AND ("chronic spontaneous urticaria"[Title/Abstract] OR "chronic idiopathic urticaria"[Title/Abstract] OR "chronic urticaria"[Title/Abstract] OR CSU[Title/Abstract] OR CIU[Title/Abstract]) AND (exacerbation[Title/Abstract] OR flare[Title/Abstract] OR flares[Title/Abstract] OR worsening[Title/Abstract] OR aggravation[Title/Abstract] OR "urticaria flare"[Title/Abstract] OR "urticarial flare"[Title/Abstract] OR "disease flare"[Title/Abstract] OR "disease exacerbation"[Title/Abstract] OR "post-injection"[Title/Abstract] OR "post injection"[Title/Abstract] OR "after injection"[Title/Abstract] OR "following injection"[Title/Abstract] OR "delayed reaction"[Title/Abstract] OR "delayed hypersensitivity"[Title/Abstract] OR "paradoxical reaction"[Title/Abstract] OR "paradoxical exacerbation"[Title/Abstract] OR "injection reaction"[Title/Abstract] OR "injection-site reaction"[Title/Abstract] OR "adverse event"[Title/Abstract] OR "adverse reaction"[Title/Abstract] OR "adverse drug reaction"[Title/Abstract] OR "treatment-emergent"[Title/Abstract]) AND english[Language]	90
DOAJ	omalizumab AND "chronic urticaria" AND flare	2
**Supplementary sensitivity search during revision**		
Scopus	TITLE-ABS-KEY ((omalizumab OR xolair OR "anti-IgE" OR "anti IgE") AND ("chronic spontaneous urticaria" OR "chronic idiopathic urticaria" OR "chronic urticaria" OR CSU OR CIU) AND (angioedema OR "angio-oedema" OR anaphylaxis OR anaphylactic OR hypersensitivity OR "injection-site urticaria" OR "injection site urticaria" OR "delayed anaphylaxis" OR "excipient reaction" OR "excipient hypersensitivity" OR "adverse effect" OR "adverse effects" OR "side effect" OR "side effects" OR "serum sickness" OR "serum sickness-like reaction" OR "serum sickness like	983

	reaction")) AND PUBYEAR < 2027 AND (LIMIT-TO (LANGUAGE , "English"))	
PubMed	(omalizumab[Title/Abstract] OR xolair[Title/Abstract] OR "anti-IgE"[Title/Abstract] OR "anti IgE"[Title/Abstract]) AND ("chronic spontaneous urticaria"[Title/Abstract] OR "chronic idiopathic urticaria"[Title/Abstract] OR "chronic urticaria"[Title/Abstract] OR CSU[Title/Abstract] OR CIU[Title/Abstract]) AND (angioedema[Title/Abstract] OR angio-oedema[Title/Abstract] OR anaphylaxis[Title/Abstract] OR anaphylactic[Title/Abstract] OR hypersensitivity[Title/Abstract] OR "injection-site urticaria"[Title/Abstract] OR "injection site urticaria"[Title/Abstract] OR "delayed anaphylaxis"[Title/Abstract] OR "excipient reaction"[Title/Abstract] OR "excipient hypersensitivity"[Title/Abstract] OR "adverse effect"[Title/Abstract] OR "adverse effects"[Title/Abstract] OR "side effect"[Title/Abstract] OR "side effects"[Title/Abstract] OR "serum sickness"[Title/Abstract] OR "serum sickness-like reaction"[Title/Abstract] OR "serum sickness like reaction"[Title/Abstract]) AND english[Language]	285
DOAJ	omalizumab AND "chronic urticaria" AND angioedema	51
	omalizumab AND "chronic urticaria" AND anaphylaxis	0
	omalizumab AND "chronic urticaria" AND hypersensitivity	0

Note. The supplementary sensitivity search was performed on 26 June 2026 during manuscript revision in response to peer-review feedback. Records retrieved refer to database outputs before deduplication and eligibility screening. Newly identified records were screened using the same predefined eligibility criteria as the original registered review. Abbreviations: CIU, chronic idiopathic urticaria; CSU, chronic spontaneous urticaria; DOAJ, Directory of Open Access Journals; IgE, immunoglobulin E; TITLE-ABS-KEY, Scopus search field for title, abstract, and keywords.

Supplementary Table S3. Structured causality-plausibility assessment for case-based reports

Author, year	Reaction phenotype	Temporal relationship	Dechallenge	Rechallenge/recurrence	Objective or clinical support	Alternative explanations	WHO-UMC-informed causality plausibility
Lestani et al., 2025	Serum sickness-like reaction	Symptoms developed a few days after the first omalizumab administration	Omalizumab was not restarted; symptoms resolved after systemic glucocorticoids	No rechallenge	Compatible SSLR phenotype; elevated CRP/ESR, low C3, isolated anti-dsDNA positivity; rheumatologic consultation excluded overt autoimmune disease	Pre-existing intermittent arthralgia and possible subclinical dysimmune background were considered	Probable/Likely
Konstantinou & Podder, 2024	Paradoxical CSU worsening with angioedema after dose escalation, coinciding with parasitic infection	CSU flare and angioedema occurred 1 week after omalizumab dose escalation to 450 mg	Omalizumab was discontinued; symptoms improved after antiparasitic treatment	No rechallenge	Positive serology for *Echinococcus granulosus* and *Toxocara canis*; complete clinical resolution after albendazole	Parasitic infection was strongly supported; spontaneous CSU flare, autoimmune disease, allergy, organophosphate exposure, and excipient hypersensitivity were considered	Possible indirect association; direct omalizumab hypersensitivity unlikely
Dies et al., 2020	Possible IgE-mediated delayed-onset anaphylaxis; severe urticarial reaction with systemic symptoms	Symptoms occurred approximately 12 hours after the first dose and 24 hours after the second dose	Omalizumab was discontinued; reaction remained persistent despite treatment	Recurrent and more severe reaction occurred after the second dose	Positive intradermal test to omalizumab; negative skin prick and patch testing; clinical and laboratory assessment performed	Primary CSU exacerbation and external factors were considered	Probable/Likely
Weiss & Smith, 2020	Serum sickness-like reaction	Symptoms developed several days after dose escalation from 150 mg to 300 mg	Omalizumab was held and then discontinued; symptoms gradually improved over several months	No rechallenge	Mild thrombocytosis; evaluation for infection, autoimmune disease, and malignancy was unrevealing	Infection, autoimmune disease, and malignancy were investigated and not supported	Probable/Likely
Eapen & Kloepfer, 2018	Serum sickness-like reaction in a pediatric patient	Symptoms developed 1 week after the second injection	Omalizumab was discontinued; malaise, joint pain, and lymphadenopathy resolved	Symptoms worsened with each subsequent monthly injection before discontinuation	Compatible SSLR phenotype with morbilliform rash, malaise, arthralgia, lymphadenopathy, headache, and mild thrombocytopenia	Infection and estrogen/progesterone sensitivity were considered; hormonal sensitivity may have contributed to baseline urticaria/angioedema	Probable/Likely
Magen & Chikovani,	Omalizumab-associated	Severe angioedema	Angioedema was absent	Recurrent angioedema	Normal C1-INH, C4, and tryptase at	Baseline CSU-associated	Probable/Likely

Author, year	Reaction phenotype	Temporal relationship	Dechallenge	Rechallenge/recurrence	Objective or clinical support	Alternative explanations	WHO-UMC-informed causality plausibility
2018	nonhistaminergic angioedema	occurred 2 days after the first injection and after subsequent injections; recurrence occurred 1 day after rechallenge	during urticaria relapse after omalizumab discontinuation	occurred after omalizumab rechallenge; later recurrence was prevented with tranexamic acid prophylaxis	baseline and during episodes; response to tranexamic acid supported nonhistaminergic pathway	angioedema, histaminergic angioedema, and complement-mediated angioedema were considered	
Ertaş et al., 2016	Urticaria flare-up, angioedema, delayed anaphylaxis, or nonspecific adverse reaction	Reactions occurred after the first, second, fifth, or sixth omalizumab dose, ranging from within 30 minutes to more than 12 hours	Omalizumab was discontinued in affected patients	Recurrence occurred in selected patients after subsequent administration	Timing-based clinical assessment; systemic manifestations in some patients, including tongue angioedema, hypotension, and severe urticarial lesions	Spontaneous CSU flare, drug ineffectiveness, and exacerbation after discontinuation of other medications were considered	Possible overall; probable/likely in selected episodes
Gönül et al., 2016	Triphasic anaphylaxis	Severe multisystem reaction occurred within minutes after the first injection, with recurrent episodes at 24 and 36 hours	No further omalizumab injections were reported; symptoms resolved after acute treatment	No rechallenge; triphasic recurrence occurred within the same post-injection episode	Multisystem anaphylaxis phenotype with laryngeal/tongue/facial angioedema, dyspnea, hypotension, tachycardia, flushing, conjunctival injection, and cyanosis; response to epinephrine and supportive treatment	Underlying CSU with angioedema was present, but immediate timing and systemic features supported anaphylaxis	Probable/Likely

Note. This table provides a structured causality-plausibility assessment informed by WHO-UMC principles. It is not a formal numerical causality score. WHO-UMC, World Health Organization–Uppsala Monitoring Centre; CSU, chronic spontaneous urticaria; SSLR, serum sickness-like reaction; CRP, C-reactive protein; ESR, erythrocyte sedimentation rate; C1-INH, C1 esterase inhibitor.
